# PFAS Mixture Composition and Internal Exposure Profiles Shape Biological Responses under Field-Realistic Exposure

**DOI:** 10.1021/acs.est.6c01726

**Published:** 2026-05-24

**Authors:** Alan M. Vajda, Jill A. Jenkins, David W. Bertolatus, Denis R. LeBlanc, Christopher J. Martyniuk, Zachary R. Hopkins, Andrea K. Tokranov, Abigail R. Dethloff, Joseph C. Tucker, Rainer Lohmann, Larry B. Barber

**Affiliations:** † 12226 University of Colorado Denver, P.O. Box 173364, Denver, Colorado 80217-3364, United States; ‡ U.S. Geological Survey, Wetland and Aquatic Research Center, Lafayette, Louisiana 70506, United States; § Adams State University, Alamosa, Colorado 81101, United States; ∥ U.S. Geological Survey, New England Water Science Center, Northborough, Massachusetts 01532, United States; ⊥ University of Florida Genetics Institute, College of Veterinary Medicine, Gainesville, Florida 32610, United States; # U.S. Geological Survey, Eastern Ecological Science Center, Kearneysville, West Virginia 25430, United States; ∇ Graduate School of Oceanography, 54083 University of Rhode Island, Narragansett, Rhode Island 02882, United States; ○ U.S. Geological Survey, Boulder, Colorado 80303, United States

**Keywords:** Per- and polyfluoroalkyl substances (PFAS), Aqueous film-forming foams (AFFF), Fathead minnow (Pimephales promelas), Reproductive biomarkers, Transcriptomics, Mixture-toxicity

## Abstract

Per- and polyfluoroalkyl substances (PFAS) occur as complex mixtures, yet mixture-dependent biological effects under environmentally realistic exposure conditions remain poorly understood. We conducted multiyear (2018, 2019, 2021) continuous-flow, field-based exposures of male fathead minnows (*Pimephales promelas*) using a low-PFAS reference well (REF; sum of measured PFAS (∑PFAS) 0.1–0.2 μg L^–1^) and PFAS-contaminated groundwater from a fire-training area (FTA) at Joint Base Cape Cod, Massachusetts. Dilution treatments enabled separation of concentration and mixture effects. Groundwater from well FTA1 was perfluorooctanesulfonate (PFOS) dominated (∑PFAS 10–31 μg L^–1^), whereas groundwater from well FTA2 had higher concentrations and was enriched in perfluorooctanoate (PFOA) and diverse precursors (∑PFAS ∼ 80 μg L^–1^). Despite comparable plasma ∑PFAS and PFOS in FTA1–100% and FTA2–50% on day-7, cumulative mortality reached approximately 35% in FTA2–50% and 17% in FTA2–100%, and secondary sex trait expression was reduced by approximately 65–80% relative to REF, whereas sperm motility effects were mixture- and time-dependent. Plasma PFAS profiles were dominated by perfluorohexanesulfonate (PFHxS), PFOS, and sulfonamide precursors. Liver transcriptomics from REF-acclimated fish revealed robust disruption of metabolic, mitochondrial, and endocrine pathways that link PFAS mixture chemistry and internal exposure profiles to organismal outcomes, and testis transcriptomics provided complementary insight into reproductive impairment. These results indicate that PFAS mixture composition and internal exposure profiles, including precursor-associated differences, are important determinants of ecotoxicological outcomes under field-realistic conditions.

## Graphical abstract

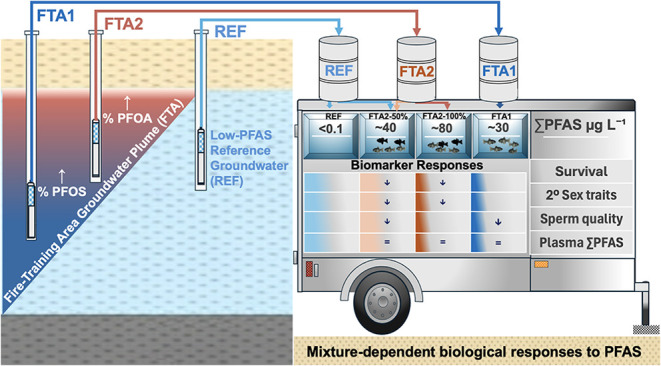

## Introduction

Per- and polyfluoroalkyl substances (PFAS) are persistent and mobile contaminants that accumulate in groundwater and surface waters.^1–3^ Aqueous film-forming foams (AFFF) used at fire-training areas (FTAs) are a major source of PFAS in groundwater. Repeated releases have generated persistent and hydrologically mobile plumes that transport PFAS into downgradient surface waters.^4–11^ As these plumes move downgradient, they can discharge to ponds, wetlands, and streams, creating localized but sustained exposure pathways for aquatic organisms at groundwater-surface water interfaces.^6,7,11,12^ In many such settings, PFAS concentrations in discharging groundwater exceed those measured in overlying surface waters by orders of magnitude.^4–8^

Although PFAS occurrence and fate are increasingly well characterized, ecotoxicological and reproductive consequences of environmentally realistic PFAS mixtures at groundwater-surface water interfaces remain less understood.^5,13–17^ AFFF-derived mixtures contain long-chain sulfonates and carboxylates, as well as fluorotelomer and sulfonamide precursors,^8,10,15,18–21^ which can undergo abiotic and biotic transformation during transport or within organisms, thereby altering mixture composition and sustaining internal exposure.^8,10,22,23^ This chemical complexity is toxicologically important because PFAS differ in toxicokinetics and toxicodynamics,^16,17^ and mixture effects may be additive, greater-than-additive, or attenuated relative to expectations from individual compounds.^24–29^

Toxicological and epidemiological evidence links PFAS to endocrine, immune, metabolic, and developmental effects in humans and wildlife.^30–35^ Fish readily accumulate PFAS from water and diet,^3,16,36,37^ and field studies have reported altered expression of genes linked to metabolism, immune function, oxidative stress, and reproduction in fish from PFAS-contaminated habitats.^38–41^ Across fish species, PFAS exposure disrupts gonadal development, steroid hormone pathways, and reproductive performance.^30,33,42–47^ Laboratory studies have shown that perfluorooctanesulfonate (PFOS) reduces sperm motility and density at 5–50 μg L^–1^,^42^ and perfluorooctanoate (PFOA) suppresses motility and alters steroidogenic signaling at <1 μg L^–1^, overlapping levels reported in contaminated groundwater.^5,8,48–51^ Interpreting biological responses to PFAS-contaminated groundwater therefore requires linking external mixture chemistry to internal exposure and associated organismal response.

Joint Base Cape Cod (JBCC), Massachusetts, USA, contains a well-characterized groundwater PFAS plume from historical AFFF use at a former FTA.^4,6,8,10,52^ The plume discharges into Ashumet Pond, where resident fish exhibit elevated PFAS tissue burdens and altered endocrine, immune, and metabolic signaling.^41^ Recent studies at JBCC demonstrated that sulfonamide precursors such as perfluorooctane sulfonamide (FOSA), perfluorohexane sulfonamide (FHxSA), and perfluorobutane sulfonamide (FBSA) are taken up by fish and may contribute to terminal perfluoroalkyl acid burdens, sustaining internal PFOS and perfluorohexanesulfonate (PFHxS) exposures.^8,10^ These features make the JBCC FTA groundwater plume well-suited for evaluating how authentic PFAS mixture composition and internal exposure relate to biological response.

This study builds on two decades of mobile laboratory experiments by the U.S. Geological Survey and the University of Colorado that quantify contaminant uptake and biological responses on-site under environmentally realistic flow-through conditions.^8,10,53–56^ In the present study, adult male fathead minnows (*Pimephales promelas*) were exposed directly to groundwater from two contrasting PFAS mixture profiles within the JBCC FTA plume and from a low-PFAS reference well to evaluate how PFAS mixture composition, precursor-associated mixture differences, and internal exposure relate to short-term organismal responses and reproductive biomarker disruption. The experimental design preserved native water chemistry while allowing simultaneous quantification of PFAS-specific water chemistry, fish plasma PFAS profiles, and fish reproductive responses including secondary sex traits, sperm motility, histopathological outcomes, and transcriptomic responses in liver and testes.

The experimental design focused on short- to intermediate-term exposures in reproductively active adult males, emphasizing organismal, reproductive, and molecular responses rather than developmental or full life-cycle toxicity. Male reproductive biomarkers were emphasized because fathead minnows provide a well-established model for detecting short-term endocrine and reproductive perturbation, while liver and testis transcriptomics provide broader mechanistic context beyond reproduction alone.^53–56^ A central feature of the design was the paired evaluation of authentic groundwater mixture chemistry and measured internal plasma PFAS profiles, allowing organismal responses to be interpreted in the context of both field-realistic external exposure and internal dose. Use of authentic groundwater rather than reconstituted mixtures preserved field realism but did not isolate specific PFAS from all potential co-occurring constituents. We hypothesized that short-term organismal and reproductive biomarker responses would align more closely with organismal internal dose and PFAS mixture composition than with external sum of measured PFAS concentration (∑PFAS). Internal concentrations integrate PFAS-specific uptake, retention, and mixture-specific exposure processes and therefore are expected to be more informative for predicting biological outcomes than water chemistry alone.

## Materials and Methods

### Study Site and Exposure Waters

Experiments were conducted at the U.S. Geological Survey (USGS) Toxic Substances Hydrology Groundwater Research Site at JBCC (Figure 1) where historical AFFF use created a PFAS contamination plume.^4,6–8,52,57–64^ Groundwater was pumped from three monitoring wells (Table S1): a low-PFAS reference well (REF) at the plume margin, and two wells within the FTA plume (FTA1 and FTA2). Site location, hydrogeology, well construction, and exposure-water sourcing are in the Supporting Information SI (Methods S1; Table S1).

**Figure 1:**
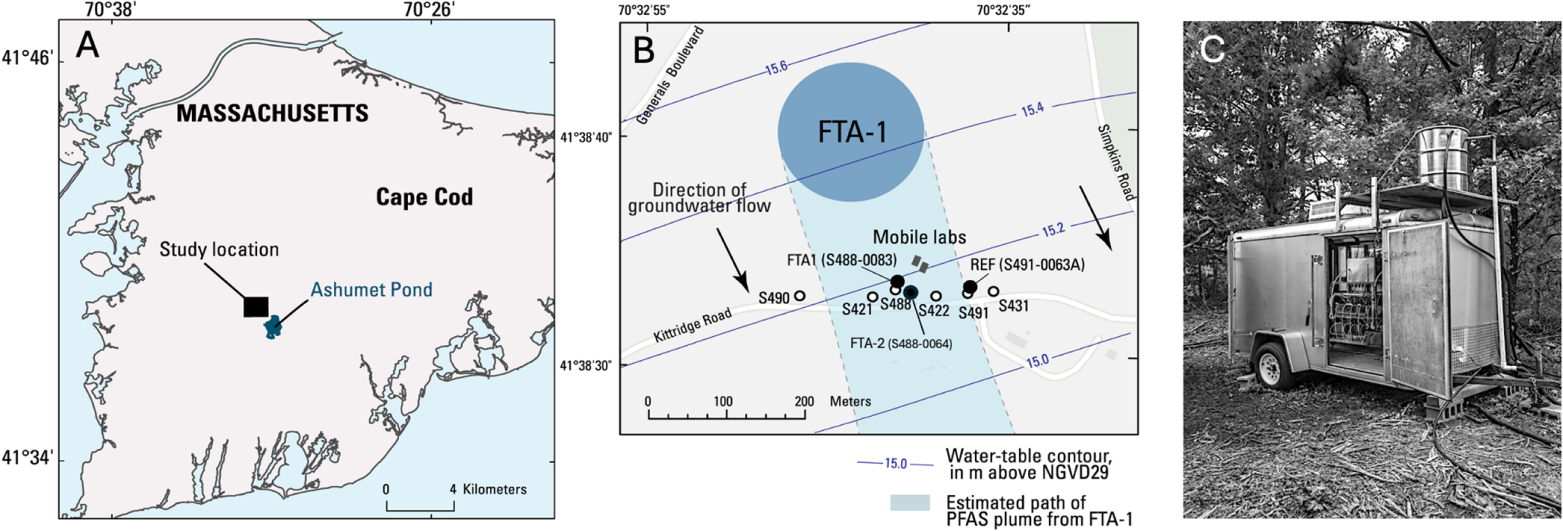
Study site overview and groundwater PFAS sources. (A) Location of study site for mobile laboratory exposure experiments conducted on Cape Cod, Massachusetts; (B) Location of PFAS-impacted groundwater contamination plume originating from a legacy aqueous film-forming foam (AFFF) fire-training area (FTA) and groundwater monitoring wells used as exposure sources (REF, low concentration reference; FTA1, intermediate concentration plume; FTA2, high concentration plume); (C) On-site, continuous-flow mobile laboratory used for fish exposures where groundwater was pumped directly from wells to holding tanks, and delivered to aquaria without filtration or chemical modification.

### Groundwater PFAS Sampling and Analysis

Groundwater was sampled at the mobile laboratory inflows. PFAS were quantified via isotope-dilution liquid chromatography-tandem mass spectrometry (LC–MS/MS).^65^ Target analytes included perfluoroalkyl sulfonates (PFSA), perfluoroalkyl carboxylates (PFCA), and AFFF-associated fluorotelomer sulfonate (FTS) and fluoroalkyl sulfonamide (FASA) precursors.^66–68^ Groundwater PFAS concentrations in intermediate dilution treatments were estimated from measured FTA and REF concentrations. Analytical procedures and analyte lists are provided in the SI (Methods S2; Tables S2–S3).

### Fish Exposure

Male fathead minnows were exposed to groundwater under continuous flow-through conditions in on-site mobile laboratories (Figure 1C). All animal care and handling procedures followed CU Denver Institutional Animal Care and Use protocol #00698 and USGS protocol USGS/WARC/LFT #2022-01. Exposure treatments in 2018 were REF and 100% FTA1 (FTA1–100%). In 2019, treatments included REF, FTA1–100%, 50% dilution of FTA1 with REF (FTA1–50%), and 25% dilution of FTA1 with REF (FTA1–25%). In 2021, treatments included REF, FTA1–100%, 100% FTA2 (FTA2–100%), and 50% dilution of FTA2 with REF (FTA2–50%). No comparable mobile laboratory exposure experiment was conducted in 2020. All exposures lasted 21 days, and in 2019 and 2021 additional 7-day exposures of REF-acclimated fish were conducted to allow separation of PFAS mixture-dependent effects from physiological stress. Mobile laboratory flow-through conditions and year-specific exposure design are detailed in the SI (Methods S3).

### Reproductive and Physiological Endpoints

Adult male fathead minnows were used because the endpoint suite emphasized male reproductive biomarkers, including secondary sex characteristics, sperm motility, testis histopathology, and testis transcriptomic responses. Endpoints were selected to capture short-term physiological, reproductive, and molecular responses under field-realistic exposure conditions rather than developmental or full life-cycle effects. At each sampling time point (initial controls, days 7 and 21 in 21-day exposures, and day-7 following the acclimation phase in REF-acclimated experiments), fish were measured for body size, gonad and liver mass, and secondary sex traits. Sperm motility and spermatogenic cell proportions were quantified, and testes were processed for histopathology.^69,70^ In 2021, liver and testis tissues from REF-acclimated fish were analyzed using microarray transcriptomics following 7-day exposures to REF, FTA1–100%, FTA2–100%, and FTA2–50%. Targeted qPCR validation of selected transcripts was not performed; therefore, transcriptomic interpretation emphasizes statistical filtering and pathway-level concordance. Endpoint analyses are described in the SI (Methods S4; Table S4).

### Plasma PFAS Measurements and Biotic Concentration Factors

Plasma collected in 2021 was used to quantify internal PFAS exposure, including selected precursor compounds, using a small-volume serum extraction method with liquid chromatography with high resolution mass spectrometry (LC-HRMS). Plasma:water biotic concentration factors (CF_B_) were calculated as plasma concentrations divided by mean groundwater concentrations for each analyte and are presented as exposure-normalized indicators of internal dose and retention rather than as steady-state bioconcentration factors. Chemical analysis descriptions and QA/QC are provided in SI (Methods S5).

### Statistical Analysis

Temporal trends in groundwater ∑PFAS during experiments were tested by simple linear regression at each site for each year. Plasma PFAS concentrations and CF_B_ were analyzed using two-way Analysis of Variance (ANOVA) with treatment and time as fixed factors, and a treatment by time interaction. Significant effects (*p* < 0.05) were further evaluated by Tukey’s post-hoc tests. Organismal indices and secondary sex traits were evaluated by one-way ANOVA (with log-transform if needed) with Tukey’s post-hoc tests, or by Kruskal–Wallis with Dunn’s post-hoc tests for ordinal scores. Statistical techniques are detailed in the SI (Methods S6).

## Results and Discussion

Mobile laboratory exposures using PFAS-contaminated groundwater at JBCC (Figure 1) showed that biological responses were more closely aligned with PFAS mixture composition and internal exposure than with groundwater ∑PFAS alone (Figure S1). Across years and sites, PFOS, PFOA, and PFHxS dominated groundwater ∑PFAS, but their relative abundance and precursor composition differed among sites (Figures 2 and S2–S3; Tables S5–S7). Plasma measurements showed site- and time-dependent differences in PFAS uptake and internal mixture composition (Figures 3 and S4–S10; Tables S8–S16). Therefore, interpretation of treatment effects in this study was based on the linkage between authentic groundwater exposure chemistry and directly measured internal PFAS burdens, rather than on external concentrations alone, while recognizing that authentic groundwater exposures do not isolate PFAS from all other possible site constituents.

**Figure 2:**
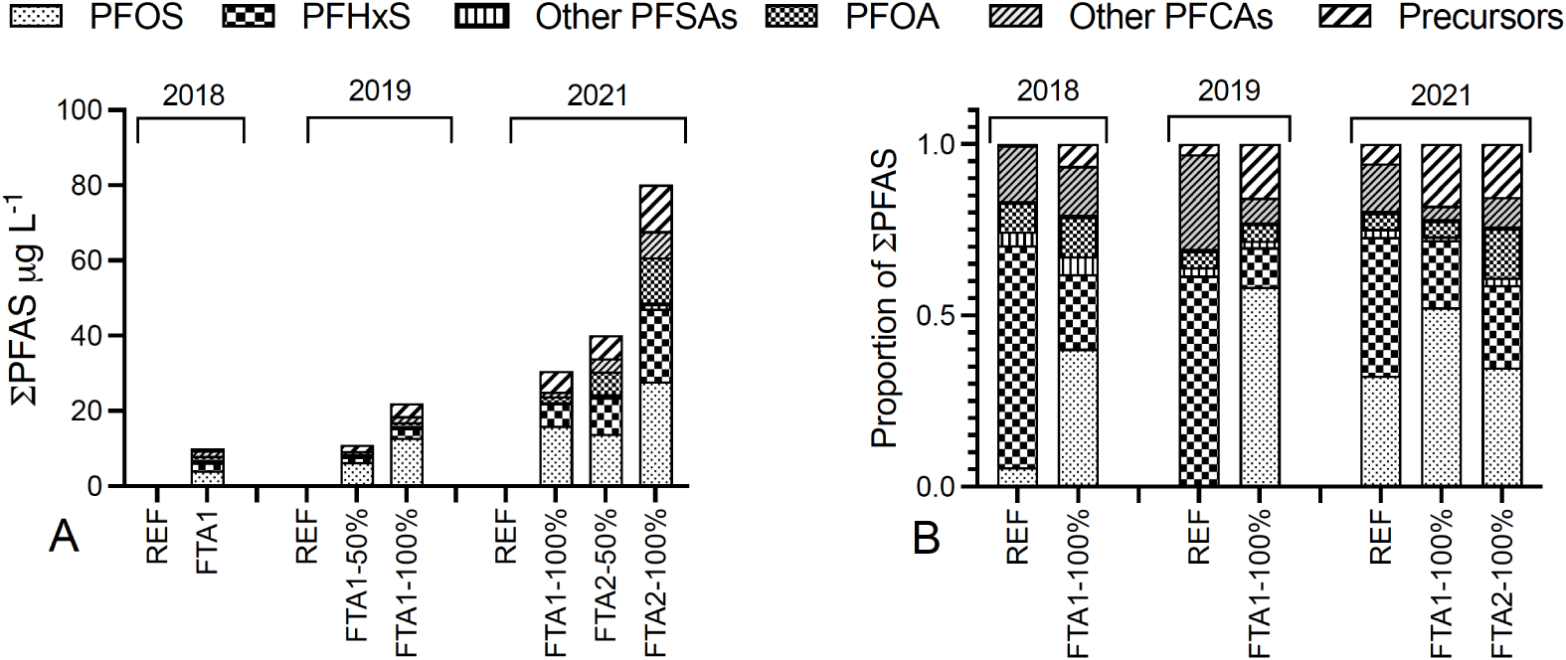
Groundwater per- and polyfluoroalkyl substances (PFAS) mixture concentrations and composition across study years. (A) PFAS concentrations (μg L^–1^) and (B) relative PFAS composition in groundwater from reference well (REF) and fire-training area wells (FTA1, FTA2) during mobile laboratory experiments conducted on Cape Cod, Massachusetts, in 2018, 2019, and 2021. [Sample sizes by year and site are provided in Tables S5 and S6; see Table S2 for PFAS abbreviations].

**Figure 3:**
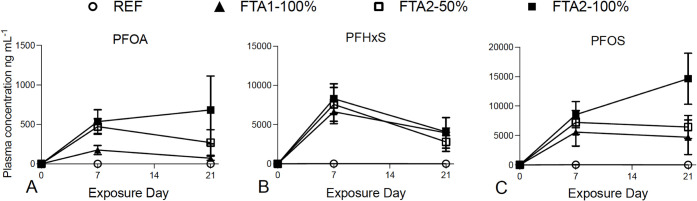
Plasma per- and polyfluoroalkyl substances (PFAS) concentrations during 2021 groundwater exposure experiments. (A) Temporal changes in fathead minnow (*Pimephales promelas*) plasma concentrations (ng mL^–1^) of PFOA, (B), PFHxS, and (C) PFOS during 2021 mobile laboratory exposures to reference well (REF) and fire-training area wells (FTA1, FTA2) conducted on Cape Cod, Massachusetts [Values are mean ± standard deviation; see Table S2 for individual PFAS abbreviations; statistical results and additional analytes are provided in Tables S8–S10].

PFOS-rich mixtures at FTA1 were associated with impaired spermatogenesis and sperm performance, whereas PFOA- and PFCA-enriched mixtures at FTA2 coincided with higher mortality, suppression of secondary sex traits, and reduced sperm performance (Figures 4 and S11; Tables S17–S18). Transcriptomic responses provided complementary mechanistic context for these apical patterns, with exploratory pathway-level responses in testis (Figures S12–S17; Tables S19–S22) and more robust liver responses across mixtures (Figures S18–S22; Tables S23–S25). Together, these findings indicate that ecotoxicological effects at contaminated sites are shaped by exposure magnitude, PFAS mixture composition, and toxicokinetic behavior.

**Figure 4:**
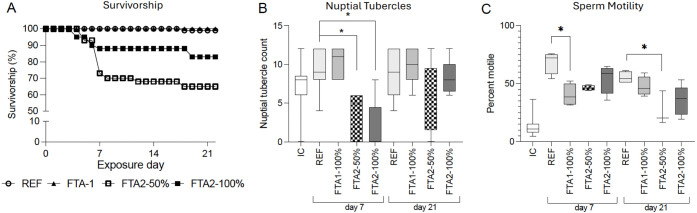
Organismal and reproductive biomarker responses to 2021 groundwater exposures. (A) Survivorship, (B) secondary sex traits, and (C) sperm motility in male fathead minnows (*Pimephales promelas*) exposed in 2021 to groundwater from a reference well (REF) and fire-training area wells (FTA1, FTA2) on Cape Cod, Massachusetts, relative to day-0 initial controls (IC), assessed on exposure day-7 and d-21. [Asterisks indicate significant pairwise differences relative to the appropriate comparison group (*p* < 0.05); sample sizes and statistical tests are provided in Tables S17–S18].

### PFAS Mixture Composition and Concentration as Drivers of Biological Outcomes

Of 34 PFAS quantified in groundwater (Table S2), 25 were detected across study years (Tables S5–S6). Full groundwater chemistry for 2018, 2019, and 2021 experiments is reported elsewhere.^66–68^ PFOS, PFOA, and PFHxS comprised 73–80% of ∑PFAS (Figures 2 and S2) and are among the PFAS most frequently associated with toxicity in fish.^16,47^ REF groundwater ∑PFAS remained low (0.1–0.2 μg L^–1^). At FTA1, ∑PFAS increased from 10 ± 2.4 μg L^–1^ (n = 13) in 2018 to 30.5 ± 4.4 μg L^–1^ (n = 15) in 2021, driven primarily by increases in PFOS and PFHxS. In 2021, FTA2 had the highest ∑PFAS (Figure S3) observed in this study (80.1 ± 6.7 μg L^–1^; n = 15).

Biological outcomes differed between sites and years in a manner consistent with PFAS mixture composition. Dilution treatments provided additional evidence that biological responses tracked changes in PFAS mixture composition rather than nominal exposure level alone. The 2019 FTA1–50% treatment had similar ∑PFAS to the 2018 FTA1–100% exposure but a higher proportional contribution of PFOS and lower contributions of PFOA and PFHxS, and this shift coincided with less severe biological effects in 2019 than 2018 (Figures 2 and S3). In 2021, FTA1–100% and FTA2–50% groundwater had comparable ∑PFAS and similar PFOS concentrations (∼14–16 μg L^–1^), yet only FTA2 exposures resulted in mortality and pronounced reproductive impairment (Figures 4 and S11; Tables S17–S18). Groundwater at FTA2 contained proportionately higher PFOA and greater compositional heterogeneity relative to FTA1 (Figure 2; Table S6). These compositional differences may have contributed to the greater effects observed at FTA2.^16,25,33^

Measured precursors also differed between sites. In 2021 groundwater, precursors constituted 18% of ∑PFAS at FTA1 and 15% at FTA2. At FTA1, the precursor pool was dominated by FHxSA (61%), whereas at FTA2 the precursors were more evenly distributed [6:2 FTS 36%; FHxSA 34%; 8:2 fluorotelomer sulfonate (8:2 FTS) and FBSA each approximately 8%] (Table S6). These differences indicate that exposure at each site was defined not only by total PFAS concentration but also by mixture composition, including precursor identity and abundance. Although such precursors may contribute to internal PFAS burdens, the present study does not directly resolve their quantitative contribution to terminal PFAS accumulation or toxicity.^8,10,22,23^

Temporal profiles of ∑PFAS during the exposure periods differed by site and year. At FTA1, ∑PFAS increased during the 2018 and 2019 experiments (*p* < 0.05; Figure S3; Table S7), thus fish experienced progressively higher external concentrations over their exposure period. In contrast, ∑PFAS remained relatively stable throughout the 2021 exposures for both the FTA1 and FTA2 treatments (Figure S3) and strengthened interpretation of mixture-composition effects. Collectively, groundwater chemistry provides the exposure context for the internal PFAS burdens and biological outcomes in exposed fish.

### Plasma PFAS Concentrations and Bioconcentration Factors

Plasma PFAS measurements quantified organism internal dose and revealed distinct site- and time-dependent differences in PFAS-specific accumulation (Figures 3 and S4–S6; Tables S8–S10). Across all treatments, PFHxS, PFOS, and FHxSA comprised 90–95% of plasma ∑PFAS (Figure S5; Table S9), consistent with slower clearance and stronger protein binding of long-chain sulfonates and precursors.^71–73^

Across treatments, plasma ∑PFAS increased rapidly between day-0 and day-7, and by day-7, plasma ∑PFAS, PFOS, and PFHxS were statistically indistinguishable among FTA1–100%, FTA2–100%, and FTA2–50% (*p* > 0.05; Figures 3, S4, and S6; Tables S8–S10). Between day-7 and day-21, plasma ∑PFAS declined significantly at both FTA1–100% and FTA2–50% (*p* < 0.05), and concentrations were statistically indistinguishable from one another at day-21 (*p* > 0.05). In contrast, plasma ∑PFAS at FTA2–100% increased significantly between day-7 and day-21 (*p* < 0.05) and was significantly higher than FTA1–100% and FTA2–50% at day-21 (*p* < 0.05; Figure S4). These divergent temporal patterns are consistent with net loss from plasma via elimination and/or redistribution to other tissues at FTA1–100% and FTA2–50%, whereas continued accumulation at FTA2–100% indicates that uptake of one or more PFAS exceeded clearance during this interval. Several additional PFAS, including perfluoropentanoate (PFPeA), perfluorohexanoate (PFHxA), perfluoroheptanoate (PFHpA), perfluorodecanoate (PFDA), perfluoroheptanesulfonate (PFHpS), and FOSA, also did not differ among treatments on day-7 (*p* > 0.05; Figure S6; Tables S8–S10). In contrast, plasma PFOA was >2.5 times higher at both FTA2–100% and FTA2–50% than at FTA1–100% (Figure 3), and PFNA and FBSA were also higher at both FTA2 treatments than at FTA1–100% (*p* < 0.05; Figure S6; Tables S8–S10). In contrast, FHxSA remained higher at FTA1–100% than at either FTA2 treatment. Plasma FBSA and perfluoropentanesulfonate (PFPeS) were significantly higher at FTA2–100% than at FTA2–50% (*p* < 0.05; Figure S6). By day-7 the treatments converged on similar plasma burdens of the dominant sulfonates, while retaining site-specific differences in PFOA, perfluorononanoate (PFNA), FBSA, and FHxSA.

Biotic concentration factors (CF_B_; plasma:water) provided a measure of PFAS-specific uptake and retention independent from external PFAS concentration (Figures S7–S8; Tables S11–S12). For PFCAs, CF_B_ increased with chain length, consistent with established relationships between carbon number, protein binding affinity, and decreased renal clearance.^71,74^ PFSA behavior was more complex. On day-7, PFHxS had the highest CF_B_ among PFSAs, consistent with both direct uptake and potential contributions from precursor transformation documented at AFFF-impacted sites.^8,10^ In contrast to laboratory uptake studies using PFAS mixtures with limited precursor inputs,^75^ the elevated and time-dependent PFHxS bioconcentration observed here under field-realistic exposure conditions is consistent with modification of apparent uptake by precursor availability and mixture heterogeneity.

By day-21, PFSA accumulation patterns had diverged among treatments. PFHxS CF_B_ decreased significantly at all sites (*p* < 0.05; Figure S8; Table S12), indicating net loss from plasma. Site-specific differences in plasma PFAS profiles and CF_B_ behavior aligned with differences in groundwater precursor composition. At FTA1, where FHxSA was the dominant precursor, CF_B_ declined on day-21 for both FHxSA and PFHxS (*p* < 0.05; Figures S7–S8; Tables S11–S12), consistent with reduced net uptake and/or increased elimination of both compounds. In contrast, at FTA2, where precursors present in the groundwater were more diverse and at higher concentrations (Tables S5–S6), FHxSA CF_B_ increased between day-7 and day-21, whereas PFHxS CF_B_ decreased sharply. This pattern indicates continued uptake of FHxSA concurrent with net loss of PFHxS from plasma, reflecting differential toxicokinetics of precursor and terminal compounds rather than direct coupling of their plasma concentrations.^10,74^

Patterns for terminal sulfonates reinforced these contrasts. By day-21, PFOS became the dominant contributor to plasma ∑PFAS in every treatment, with its proportional contribution increasing from 29 to 41% at FTA1–100%, from 36 to 42% at FTA2–50%, and from 41 to 57% at FTA2–100% (Figure 3). This shift occurred even though plasma PFOS concentrations increased only at FTA2–100%, while remaining statistically unchanged at FTA1–100% and FTA2–50% (Tables S8–S10). Over the same period, plasma PFHxS concentrations declined significantly across all treatments (*p* < 0.05; Figure 3; Tables S8–S10). Thus, PFOS became the dominant plasma PFAS at FTA1–100% and FTA2–50% primarily because other PFAS, notably PFHxS and FHxSA, declined, whereas at FTA2–100% its dominance reflected both an absolute increase in PFOS concentration and concurrent declines in other PFAS. Although precursors to PFOS and PFHxS were present in the exposure mixtures, divergent trends in these terminal sulfonates indicate that internal PFSA distributions reflected multiple toxicokinetic processes, including differential elimination, plasma protein binding, and tissue redistribution, rather than precursor contribution alone.^3,23,37,73–76^

The sulfonamide precursor FOSA further demonstrates complex precursor-product relationships. FOSA had the highest CF_B_ of all quantified plasma PFAS in every treatment (Figure S7) and exhibited site-specific temporal behavior (Tables S11–S12). By day-21, plasma FOSA concentrations declined at FTA1–100% with no change in PFOS, increased at FTA2–50% with no change in PFOS, and increased at FTA2–100% alongside increased PFOS (Figures 3 and S6; Tables S8–S10). These contrasting patterns suggest that conversion, elimination, and plasma-protein partitioning interact in a site- and treatment-dependent manner, and that no single mechanism explains responses from all groups.

Additional precursor classes not quantified in this study are likely present at this AFFF-impacted site, so the relative contributions of measured versus unmeasured precursors to internal PFAS mixture composition remain uncertain.^10,15,18–21^ These internal PFAS patterns indicate that toxicological interpretation depends not only on external mixture composition, but also on PFAS-specific uptake, precursor-associated inputs, binding interactions, and redistribution processes that shape the biologically relevant internal mixture. Although precursor profiles differed clearly between sites and several precursor compounds were detected in plasma, the present study does not directly quantify in vivo biotransformation rates or isolate the contribution of measured precursors to terminal PFAS burdens and toxicity.

#### Site-Specific Biomarker Responses Reflect Concentration and Compositional Differences

Across years, groundwater exposures produced reproducible but treatment-specific reproductive impairments in adult male fathead minnows. In 2021, mortality occurred only at FTA2–50% (∼40 μg L^–1^ ∑PFAS) and FTA2–100% (∼80 μg L^–1^ ∑PFAS), with cumulative mortality reaching 35% and 17%, respectively, whereas FTA1–100% produced no mortality despite similar PFOS concentrations to FTA2–50%. Sperm motility effects also differed by treatment and sampling time (Figures 4, S1, and S11; Tables S17–S18). REF differed slightly from day-0 initial controls (IC), but effects were small and not indicative of adverse fish health, i.e., increased body mass, sperm motility, and secondary sex traits. Because this study focused on male reproductive biomarkers, the results should not be assumed to capture all sex-specific responses to PFAS-contaminated groundwater, and female responses warrant future investigation.

#### FTA1 Exposures: Cellular Effects without Organismal Impairment

In 2018, exposure to FTA1–100% groundwater (∑PFAS ∼ 10 μg L^–1^) did not alter survivorship, secondary sex traits, hepatosomatic index (HSI), gonadosomatic index (GSI), or hepatic vitellogenin mRNA (Tables S17–S18). However, histopathology results indicated reduced sperm abundance and elevated spermatogonial apoptosis, demonstrating that cellular impairment can occur in the absence of gross organismal effects. In 2019, groundwater ∑PFAS at FTA1 doubled, yet no significant changes in survivorship, secondary sex traits, HSI, GSI or histopathology were detected. These contrasts aligned with a compositional shift in groundwater chemistry in which PFOA and PFHxS declined while the PFOS fraction increased from 40% to 52%, reinforcing the importance of PFAS mixture composition to organismal responses.^16,47^ Sperm performance was not evaluated in 2018; in 2019, motile and progressive sperm fractions were reduced at day-7 but recovered by day-21, indicating transient functional impairment (Tables S17–S18).

#### FTA2 Exposures: Stronger Organismal Response to PFOA- and PFCA-Enriched Mixtures

In 2021, exposures at FTA2 produced a qualitatively different hazard profile than FTA1 mixtures. Early impairment of sperm performance occurred only at FTA1–100%, whereas delayed impairment at day-21 occurred only at FTA2–50% (Figure 4; Tables S17–S18). No significant motility reduction was detected in fish exposed to FTA2–100% despite 2-fold higher ∑PFAS relative to FTA2–50%. Because early mortality occurred at both FTA2 treatments, day-21 sperm measurements reflect surviving fish and may underestimate effects in the fully exposed cohort; they therefore should not be interpreted as a simple monotonic continuation of initial exposure severity.

Flow cytometry showed no significant differences in the proportions of sperm, spermatids, or diploid cells across treatments, indicating functional impairment of mature sperm rather than reduced spermatogenesis.^77–79^ These patterns demonstrate that sperm function depends on internal toxicokinetics and toxicodynamics and does not scale predictably with external concentrations. This aligns with evidence that PFOA and PFOS impair sperm function through effects on membrane fluidity, mitochondrial energetics, calcium signaling, redox balance, and oxidative stress without necessarily altering germ cell proportions, providing a plausible mechanistic basis for reduced sperm performance in the absence of marked changes in spermatogenic cell distributions.^25,33,51,80,81^

#### Mortality and Secondary Sex Traits Track Mixture Composition and Energetic Stress

Mortality occurred only in 2021 at FTA2–100% and FTA2–50% and was absent in FTA1–100% despite comparable ∑PFAS between FTA2–50% and FTA1–100% (Figures 4 and S11; Table S18). The ∑PFAS associated with elevated mortality in 2021 (FTA2–50% ∼ 40 μg L^–1^; FTA2–100% ∼ 80 μg L^–1^) were the highest measured in this study yet remained orders of magnitude below the 50% lethal concentration (LC_50_) for individual PFAS.^16,27^ Mortality occurred primarily during the first 7 days (Figure 4A) and coincided with abnormal swimming behavior observed during daily checks, including reduced buoyancy and impaired fin movement, consistent with acute physiological stress. Secondary sex traits were suppressed only at FTA2–100% and FTA2–50% (Figure 4B). Plasma PFAS profiles were consistent with these patterns, with fish at FTA2–100% and FTA2–50% exhibiting proportionally greater PFOA accumulation than at FTA1–100% during early exposure (Figures 3 and S5–S6; Tables S8–S10). Although absolute differences in PFOA plasma concentration were modest, enrichment of PFOA within a more compositionally complex mixture coincided with early mortality and suppression of secondary sex traits, whereas continued accumulation at FTA2–100% beyond day-7 did not result in additional mortality.

Together, these results support an interpretation in which early mixture-driven energetic constraint, rather than exceedance of acute single-compound toxicity thresholds, contributed to observed organismal responses under field-realistic exposure conditions.^16,82–84^

#### Conventional Biomarkers Lacked Sensitivity

Conventional biomarkers of endocrine disruption (e.g., GSI, vitellogenin mRNA) showed limited sensitivity relative to sperm function and histopathology despite histological and functional impairments (Tables S17–S18), consistent with earlier studies at environmentally relevant PFAS concentrations.^43,47,85,86^ These findings indicate that conventional biomarkers may underestimate ecotoxicological risk, while plasma PFAS mixture composition, sperm performance, and testis histopathology more reliably capture mixture-specific reproductive toxicity.

#### Internal Dose and Nonlinear Exposure–Response Relationships

Although groundwater ∑PFAS differed 2-fold between FTA2–100% and FTA2–50% treatments (Figure 2; Tables S5–S6), both exposures produced comparable mortality during the first 7 days (Figure 4). On day-7, plasma ∑PFAS, PFOS, PFHxS, and additional PFAS [PFPeA, PFHxA, PFHpA, PFDA, perfluoroheptanesulfonate (PFHpS), FOSA] were statistically indistinguishable among treatments (*p* > 0.05; Figures 3 and S6; Tables S8–S10), whereas plasma PFOA, PFNA, and FBSA also were higher in both FTA2 treatments than FTA1–100% (*p* < 0.05; Figure S6; Tables S8–S10). These patterns indicate that early apical responses were associated less with total plasma burden than with treatment-specific internal composition and the timing of exposure during a stress-sensitive interval.

After day-7, no additional mortality occurred, even as plasma profiles diverged. Between day-7 and day-21, PFHxS declined significantly at all sites, whereas PFOS and PFNA increased only at FTA2–100%, and FBSA increased at both FTA2 treatments (*p* < 0.05; Figures 3 and S6; Tables S8–S10). In contrast, plasma PFOA did not increase between day-7 and day-21 at either FTA2 treatment (Figure 3; Tables S8–S10), indicating that internal PFOA burdens approached an early plateau in the exposure period.^75^ These patterns are consistent with saturation of binding interactions and exchange equilibria that can limit further increases in plasma with higher external concentrations,^74,75^ although other processes such as tissue redistribution may also contribute. Thus, later divergence in internal burden did not translate into additional mortality, suggesting that the critical window for organismal impairment occurred early in exposure. The timing of mortality therefore aligned with the early exposure interval in which the two FTA2 treatments shared similar PFSA burdens and shared elevated PFOA and PFNA relative to FTA1, whereas later increases in PFOS, PFNA, and FBSA at FTA2–100% did not produce additional apical effects.

Reproductive effects were also nonmonotonic. Sperm impairment at FTA2 was detected only in FTA2–50% on day-21 and not at FTA2–100%, despite the higher external concentration and greater later divergence in internal burden at FTA2–100% (Figures 3 and S5; Tables S8–S10). These patterns indicate that initiation of toxicity was not limited by later PFOS accumulation alone. Because early mortality occurred at both FTA2 treatments, day-21 sperm measurements likely reflect surviving fish and may therefore underestimate reproductive impairment in the full exposed cohort. Taken together, these results are consistent with mixture-dependent toxicokinetics,^16,25,33^ early threshold-like mortality, and survivor bias in later reproductive measurements rather than with a simple monotonic concentration–response relationship.

### Role of Acclimation and Multiple Stressors in Modulating PFAS Toxicity

Handling and osmotic transfer stress modified PFAS toxicokinetics and organismal sensitivity. Fish were transported in buffered freshwater with higher ionic strength than the low-conductivity groundwater at the REF and FTA sites. Rapid transfer into low-solute water can disrupt ionoregulation, elevate cortisol, increase reactive oxygen species, and reduce ATP availability, impairing survival and reproduction.^82,83,87^

In the nonacclimated 2021 cohort, the combination of transport and immediate exposure to PFAS-contaminated groundwater coincided with substantial mortality at FTA2–100% (17%) and FTA2–50% (35%) and reduced nuptial tubercles at day-7 (Figure 4; Tables S17–S18). No comparable effects occurred in REF fish, which experienced the same handling and low-conductivity transfer conditions and therefore served as a transfer-stress control in the absence of elevated PFAS. Together, these results support an interpretation in which transfer-associated physiological stress reduced tolerance to PFAS mixtures perhaps by increasing energetic demand and lowering the threshold for mixture-driven toxicity during the early exposure period.

Acclimation eliminated these early effects. When fish were acclimated in REF for 13 days prior to a 7-day exposure, mortality and suppression of secondary sex traits were not observed in any treatment (Tables S17–S18). PFOS-dominated FTA1 mixtures did not induce mortality in 2018, 2019, or 2021 (Figure S11), whereas mortality occurred only at FTA2 (100% and 50%) in the nonacclimated cohort, indicating heightened sensitivity to the mixture under transport and transfer conditions. Sperm performance followed similar patterns. In nonacclimated cohorts, motile and progressive sperm fractions were significantly reduced at FTA1–100% on day-7 in both 2019 and 2021 but recovered by day-21, and no sperm impairment occurred in nonacclimated REF or acclimated treatments. These results indicate that transport and transfer stress can increase sensitivity to PFAS in the early exposure period but did not produce adverse outcomes in the absence of PFAS mixtures.

Acclimation also altered plasma PFAS profiles. Internal burdens decreased in acclimated fish at FTA1–100%, remained similar at FTA2–50%, and increased at FTA2–100% (Figure S9). Despite this increase, acclimated fish at FTA2–100% experienced no mortality, indicating that recovery from transfer stress increased tolerance to PFAS mixtures independent of internal PFAS burden. Mixture composition also shifted following acclimation. Acclimated FTA1 fish contained proportionally more precursors and less PFOS, whereas acclimated FTA2–100% fish contained proportionally more PFOS and less PFHxS (Figures S9–S10). These shifts are consistent with osmotic preconditioning altering uptake and possibly precursor-related processes, although mechanisms cannot be separated with the current data.

### Transcriptomic Evidence of Pathway Disruption and Mechanistic Linkages

Liver and testis transcriptomics were evaluated in 2021 male fathead minnows acclimated in REF for 13 days prior to a 7-day exposure, minimizing physiological stress associated with immediate transfer and enabling clearer resolution of PFAS mixture-dependent molecular responses.

#### Testis Transcriptomic Responses Align with Reproductive Impairment

Testis transcriptomics revealed coordinated pathway-level disruption of mitochondrial, endocrine, and immune pathways in REF-acclimated fish exposed to FTA2–100% and FTA2–50% groundwater (Figures S12–S17; Tables S19–S22), consistent with observed effects on sperm performance and secondary sex traits. Of the 11,387 detected transcripts, none met false discovery rate (FDR)-adjusted significance; therefore, these data were interpreted conservatively and at the pathway level only. Using an unadjusted threshold of *p* < 0.05, 1,373, 1,546, and 1,885 transcripts were nominally different between FTA2–100% vs REF, FTA2–50% vs REF, and FTA2–100% vs FTA2–50%, respectively (Figure S17). Transcriptomic divergence between the two FTA2 treatments exceeded that observed between either treatment and REF, indicating sensitivity to internal exposure context and mixture composition beyond the presence of PFAS alone.

Plasma PFAS data from the same fish confirmed higher total burdens at FTA2–100% than FTA2–50%, with modest shifts in PFOS and PFHxS proportions (Figure S9; Tables S13–S16), providing chemical context for transcriptional differences. Pathway enrichment analyses identified disruption of mitochondrial bioenergetics, DNA damage response, and hormone-responsive signaling, consistent with chain-length–dependent differences in PFAS toxicodynamics reported for PFHxA versus PFHxS in fish.^88^ KEGG and Hallmark analyses (SI Method S4) highlighted oxidative phosphorylation, p53 signaling, and peroxisome proliferator-activated receptor (PPAR) pathways (Figures S12–S14), consistent with mitochondrial stress and altered metabolic regulation. Androgen- and estrogen-responsive gene sets were perturbed (Tables S19–S22), aligning with suppressed secondary sex traits and reduced sperm motility and consistent with verified in vivo estrogenic activity reported in fish.^89^

Immune-related pathways were among the most consistently affected domains. Both FTA2 treatments enriched RIG-I–like receptor and Toll-like receptor signaling converging on IL-6, IL-8, and TNFα (Figures S15–S16), consistent with PFAS-induced innate immune activation and mitochondrial-immune crosstalk reported in fish exposed to short-chain PFAS, including PFBA and PFBS.^90^ Additional enrichment of HIF-1 and ERK pathways suggested hypoxic stress and altered cellular signaling, particularly at FTA2–100%. Overall, testis transcriptomic responses were directionally consistent across FTA2 treatments, indicating sensitivity to subtle shifts in mixture composition and internal dose rather than simple proportional concentration effects. These responses occurred in acclimated fish with limited apical impairment, contrasting with the higher mortality and sperm dysfunction observed in nonacclimated cohorts. Accordingly, the testis data set provides exploratory mechanistic context for reproductive impairment, but it should not be interpreted as transcript-level confirmation of specific molecular drivers.

Although the transcriptomic data do not establish a direct causal pathway from innate immune signaling to reduced sperm motility or suppressed secondary sex traits, the combined enrichment of mitochondrial, inflammatory, hypoxia-related, and hormone-responsive pathways is consistent with impaired energetic support and endocrine regulation in the testis. Such coordinated disruption provides mechanistic context for reduced sperm performance and androgen-dependent trait expression, even though the proximate drivers of specific apical endpoints cannot be resolved from transcriptomic data alone.

#### Liver Transcriptomic Responses Reveal Central Metabolic and Endocrine Targets of PFAS Mixtures

Liver transcriptomics exhibited a robust and statistically resolved response to PFAS mixtures under acclimated exposure conditions (Figures S18–S22; Tables S23–S25), in contrast to the FDR-limited responses observed in the testis. Liver pathway interpretation is supported by a substantial set of FDR-significant transcripts prior to enrichment analysis. Using FDR = 0.05, 1,219 transcripts differed between FTA1–100% and REF, 1,838 between FTA2–100% and REF, and 1,798 between FTA2–50% and REF. An UpSet analysis demonstrated substantial overlap among treatments, with the largest number of unique differentially expressed transcripts associated with FTA2–100% exposure (Figure S18), indicating both conserved and mixture-specific hepatic responses.

Pathway enrichment analyses revealed coordinated disruption of cytoplasmic translation and mitochondrial energy metabolism, including oxidative phosphorylation, respiratory chain complex assembly, ATP synthesis–coupled electron transport, fatty acid metabolism, peroxisome function, and xenobiotic metabolism (Table S23). Hallmark analyses showed enrichment of oxidative phosphorylation, adipogenesis, and fatty acid metabolism in FTA1–100% relative to REF (Figure S19), whereas FTA2–100% had additionally enriched reactive oxygen species–associated pathways and exhibited a pronounced oxidative phosphorylation deficiency signature characterized by widespread transcript up-regulation (Figures S20–S22).

Across all liver comparisons, common enriched pathways included oxidative phosphorylation, lipid and atherosclerosis, chemical carcinogenesis–reactive oxygen species, nonalcoholic fatty liver disease, mRNA surveillance, antifolate resistance, and estrogen signaling (Figure S22; Table S25), consistent with PFAS modulation of estrogen signaling in fish.^91^ Analysis of upstream chemicals, drugs, and toxicants in iPathwayGuide showed a similar treatment contrast (Figures S23–S25; Tables S26–S28). A shared core of predicted signatures was recovered across treatments, but enrichment was broader in FTA2 than in FTA1. These results are consistent with site-specific mixture effects not explained by groundwater ∑PFAS alone, but they do not identify the causal chemicals present in exposure water. The consistency of these pathways across FTA1 and FTA2 indicates that hepatic metabolic and endocrine disruption represents a conserved response to PFAS mixtures, whereas the greater magnitude of responses at FTA2 aligns with higher internal PFOA burdens and greater mixture heterogeneity documented in plasma PFAS profiles.

Enrichment of estrogen-signaling pathways in liver transcriptomes (Figure S22; Table S23) indicates disruption of hepatic-endocrine regulation and steroid-responsive processes, complementing hormone-responsive perturbations observed in the testis. Together with mitochondrial and lipid-metabolic disruption, these hepatic responses provide a mechanistic framework linking PFAS mixture chemistry and toxicokinetics to organismal outcomes, including reduced survival and suppression of secondary sex traits. Although liver transcriptomics reflect acclimated, short-term exposures and are not directly comparable in magnitude to responses observed in nonacclimated cohorts, they provide critical mechanistic context for interpreting mixture-dependent toxicity under field-realistic exposure conditions. Across tissues, these transcriptomic responses support a mechanistic framework in which PFAS mixture composition and internal exposure profiles perturb mitochondrial energetics, endocrine-responsive signaling, and immune pathways that together help explain reduced survival, suppression of secondary sex traits, and altered sperm performance.

### Linking FTA Groundwater Plumes to Ashumet Pond Surface Water and Resident Biota

Ashumet Pond lies directly downgradient from the FTA groundwater contamination plume and receives sustained discharges of PFAS-contaminated groundwater.^6^ Surface water PFAS in the pond (∼200 ng L^–1^) is lower than source groundwater (tens of μg L^–1^), yet mixture composition remains enriched in PFHxS, PFOS, and PFOA, and nearly half of the PFAS mass consists of precursor compounds.^6,52^ These precursors undergo microbial and abiotic transformation to terminal sulfonates, particularly PFHxS and PFOS,^9^ sustaining long-chain PFAS in surface waters despite hydrologic dilution.

Biota in Ashumet Pond reflect this exposure. Walsh et al.^41^ reported that largemouth bass and banded killifish contained greatly elevated plasma PFAS, with PFOS in bass exceeding the reference site by more than 650-fold. Histological and transcriptomic analyses of resident fish revealed oxidative stress, immune modulation, and endocrine disruption, including enrichment of mitochondrial and hormone-related pathways. These molecular signatures parallel those in groundwater-exposed mobile laboratory fish, supporting the predictive value of PFAS mixture composition and precursor-associated internal dose at the population scale.^41^

Groundwater chemistry determines the PFAS profile delivered to the pond, and transformation processes can sustain PFOS and PFHxS in surface waters.^6^ Fish inhabiting Ashumet Pond exhibit similar mechanistic signatures of mitochondrial dysfunction, immune activation, and endocrine disruption that were identified in these experimental exposures. Thus, the mobile laboratory model provides a tractable framework for interpreting exposure-response relationships while also reproducing the exposure conditions shaping biological responses in a real, PFAS-impacted ecosystem. At the same time, interpretation of these field-realistic exposures remains bounded by the chemical complexity of the source groundwater.

Contaminated groundwater typically contains mixtures of co-contaminants, including metals, perchlorate, munitions residues, chlorinated solvents, and 1,4-dioxane.^6,7,60,61,64,92^ Previous characterization of the JBCC FTA groundwater indicated low dissolved organic carbon and relatively minimal co-occurring contaminants, suggesting lower overall chemical complexity than is typical of many contaminated groundwater systems.^8,66,68^ We did not quantify all possible co-contaminants, nontarget PFAS, or transformation products, so some portion of the observed responses could reflect combined influences of measured and unmeasured constituents.^93^ Consistent with that uncertainty, liver upstream chemical, drug, and toxicant analysis also suggested broader chemical-stress signatures in FTA2 than in FTA1 (Figures S23–S25; Tables S26–S28), but this pattern cannot distinguish effects of unmeasured co-contaminants from effects of site-specific differences among the measured contaminant mixtures. This limitation is inherent to environmentally realistic exposure designs and indicates that our conclusions should be interpreted as identifying PFAS mixture composition as an important explanatory factor rather than establishing PFAS-only causality. Follow-up studies using reconstituted PFAS mixtures based on the measured groundwater profiles would be valuable for separating PFAS-driven effects from contributions of co-occurring constituents and for more directly testing causal roles of specific PFAS classes and precursor-associated mixture differences.

#### Environmental Implications for PFAS Mixture Risk Assessment

Under field-realistic exposure conditions, external PFAS concentration remained an important component of exposure characterization, but aggregate groundwater ∑PFAS did not fully account for the observed biological response patterns, which were better interpreted in the context of mixture composition and measured internal exposure. These conclusions are drawn from short- to intermediate-term exposures in adult males and should be interpreted within that biological scope. Biological outcomes more closely tracked PFAS mixture composition, internal exposure profiles, and physiological state, with elevated PFOA and other PFCA burdens distinguishing the more severe responses observed at FTA2 from those at FTA1. Consistent with emerging ecological risk frameworks,^16^ these findings indicate that hazard within AFFF-impacted waters is shaped by a broader mixture context in which co-occurring PFCAs, precursors, and PFAS-specific toxicokinetics influence biologically relevant internal dose. Transcriptomic enrichment of pathways related to mitochondrial dysfunction, innate-immune activation, and hormone-responsive signaling was consistent with the observed organismal impairments. Although single-compound causality cannot be resolved in this field-realistic design, the combined chemical, biological, and molecular evidence indicates that PFAS mixture composition and internal exposure are important explanatory factors in the observed responses, supporting ecological risk assessment approaches that more explicitly consider mixture composition, internal exposure, precursor-associated patterns, and co-occurring stressors.

## Supplementary Material





## Notes

Supplemental methods describing the study site and exposure waters, groundwater chemistry, mobile laboratory exposure conditions, biomarker analyses, plasma PFAS extraction and biotic concentration factor calculations, statistical analyses, and expanded results; 25 supplemental figures summarizing organismal endpoints, groundwater and plasma PFAS concentrations and composition, plasma biotic concentration factors, mortality, and testis and liver transcriptomic responses; 28 supplemental tables providing well construction details, PFAS abbreviations and physicochemical properties, biomarker scoring criteria, groundwater and plasma PFAS data, biotic concentration factors, statistical outputs, organismal biomarker summaries, and transcriptomic pathway, biological process, and upstream signature analyses; Figure S1: summary of organismal endpoints across treatments; Figure S2: PFOA, PFHxS and PFOS concentrations across treatments; Figure S3: temporal changes in in PFAS groundwater concentrations; Figure S4: Plasma ∑PFAS temporal dynamics; Figure S5: Plasma PFAS mixture composition; Figure S6: Plasma PFAS concentration over time; Figure S7: Plasma PFAS CF_B_ day not acclimated; Figure S8: Plasma PFAS CF_B_ day not acclimated over time; Figure S9: Plasma PFAS day-7 acclimated vs not acclimated; Figure S10: Plasma PFAS day-7 CF_B_ acclimated vs not acclimated; Figure S11: Mortality patterns of male fathead minnows; Figure S12: Testis microarray UpSet diagram; Figure S13: Testis microarray hallmark pathways FTA2–100% vs REF; Figure S14: Testis microarray hallmark pathways FTA2–100% vs FTA2–50%; Figure S15: Testis microarray RIG-1 pathway; Figure S16: Testis microarray Toll-like receptor pathway; Figure S17: Testis transcriptomic responses in REF-acclimated fish exposed to FTA2; Figure S18: Liver microarray UpSet diagram; Figure S19: Liver microarray hallmark pathways FTA1–100% vs REF; Figure S20: Liver microarray hallmark pathways FTA2–100% vs REF; Figure S21: Liver microarray oxidative phosphorylation (OXPHOS) deficiency pathway; Figure S22: Liver microarray estrogen signaling pathway; Figure S23: Liver microarray upstream signatures FTA1–100% vs REF; Figure S24: Liver microarray upstream signatures FTA2–100% vs REF; Figure S25: Liver microarray upstream signatures FTA2-50% vs. REF (PDF)
Liver microarray upstream signatures FTA2–50% vs REF; Table S1: Well construction; Table S2: PFAS abbreviations; Table S3: PFAS physicochemical properties; Table S4: Key to biomarkers; Table S5: Groundwater PFAS extended; Table S6: Groundwater PFAS summary; Table S7: Groundwater PFAS over time Linear Regression; Table S8: Fish plasma PFAS extended; Table S9: Fish plasma PFAS summary; Table S10: Fish plasma PFAS ANOVA for treatment x time; Table S11: Fish plasma CF_B_; Table S12: Fish plasma CF_B_ ANOVA for treatment x time; Table S13: Fish plasma PFAS on day-7 in acclimated fish extended; Table S14: Fish plasma PFAS on day-7 in acclimated fish summary; Table S15: Fish plasma CF_B_ on day-7 in acclimated fish; Table S16: Fish plasma CF_B_ on day-7 in acclimated fish ANOVA for treatment x time; Table S17: Mobile lab fish biomarkers extended; Table S18: Mobile lab fish biomarkers summary; Table S19: Testis microarray enriched pathways; Table S20: Testis microarray hallmark signatures; Table S21: Testis microarray biological processes; Table S22: Testis microarray top biological processes; Table S23: Liver microarray enriched pathways; Table S24: Liver microarray biological processes; Table S25: Liver microarray top biological processes; Table S26: Liver microarray upstream signatures FTA1–100% vs REF; Table S27: Liver microarray upstream signatures FTA2–100% vs REF; Table S28: Liver microarray upstream signatures FTA2–50% vs REF (XLSX)
