# Supplementary material for: PFAS Mixture Composition and Internal Exposure Profiles Shape Biological Responses under Field-Realistic Exposure
Source: Environ Sci Technol. 2026 May 24;60(22):15656–68. doi: 10.1021/acs.est.6c01726 (PMC13262041; doi:10.1021/acs.est.6c01726)

Supporting Information

PFAS Mixture Composition and Internal Exposure Profiles Shape Biological Responses under Field-Realistic Exposure

Alan M. Vajda[1*], Jill A. Jenkins[2], David W. Bertolatus[3], Denis R. LeBlanc[4], Christopher J. Martyniuk[5], Zachary R. Hopkins[6], Andrea K. Tokranov[7], Abigail R. Dethloff[8], Joseph T. Tucker[9], Rainer Lohmann[10], Larry B. Barber[11]

[1*] University of Colorado Denver, Denver, CO 80217-3364, USA

[2] U.S. Geological Survey, Wetland and Aquatic Research Center, Lafayette, LA, 70506, USA

[3] Adams State University, Alamosa, CO 81101 USA

[4] U.S. Geological Survey, New England Water Science Center, Northborough, MA 01532, USA

[5] University of Florida Genetics Institute, College of Veterinary Medicine, Gainesville, FL 32610, USA

[6] U.S. Geological Survey, Eastern Ecological Science Center, Kearneysville, WV 25430, USA

[7] U.S. Geological Survey, New England Water Science Center, Northborough, MA 01532, USA

[8] University of Florida Genetics Institute, College of Veterinary Medicine, Gainesville, FL 32610, USA

[9] University of Colorado Denver, P.O. Box 173364, Denver, CO 80217-3364, USA

[10] Graduate School of Oceanography, University of Rhode Island, Narragansett, RI 02882, USA

[11] U.S. Geological Survey, Boulder, CO 80303, USA

*Corresponding Author

Summary of Supporting Information:
45 pages
25 figures
28 tables

Supporting Information

SI Methods

S1. Study Site and Exposure Waters
S2. Groundwater PFAS and Co-Occurring Contaminant Analytical Chemistry
S3. Mobile Laboratory Fish Exposure Conditions
S4. Fish Biomarker Analysis
S5. Plasma PFAS Extraction and Analysis: Biotic Concentration Factors
S6. Statistical Analyses
S7. Results

SI Figures

Figure S1. Summary of organismal endpoints across treatments
Figure S2. PFOA, PFHxS and PFOS concentrations across treatments
Figure S3. Temporal changes in PFAS groundwater concentrations
Figure S4. Plasma ∑PFAS temporal dynamics
Figure S5. Plasma PFAS mixture composition
Figure S6. Plasma PFAS concentration over time
Figure S7. Plasma PFAS CF_B day not acclimated
Figure S8. Plasma PFAS CF_B day not acclimated over time
Figure S9. Plasma PFAS day 7 Acclimated vs. Not Acclimated
Figure S10. Plasma PFAS day 7 CF_B Acclimated vs. Not Acclimated
Figure S11. Mortality patterns of male fathead minnows
Figure S12. Testis microarray UpSet diagram
Figure S13. Testis microarray hallmark pathways FTA2-100% vs. REF
Figure S14. Testis microarray hallmark pathways FTA2-100% vs. FTA2-50%
Figure S15. Testis microarray RIG-1 pathway
Figure S16. Testis microarray Toll-like receptor pathway
Figure S17. Testis transcriptomic responses in REF-acclimated fish exposed to FTA2
Figure S18. Liver microarray UpSet diagram
Figure S19. Liver microarray hallmark pathways FTA1-100% vs. REF
Figure S20. Liver microarray hallmark pathways FTA2-100% vs. REF
Figure S21. Liver microarray Oxidative phosphorylation (OXPHOS) deficiency pathway
Figure S22. Liver microarray estrogen signaling pathway
Figure S23. Liver microarray upstream signatures FTA1-100% vs. REF
Figure S24. Liver microarray upstream signatures FTA2-100% vs. REF
Figure S25. Liver microarray upstream signatures FTA2-50% vs. REF

SI Tables

Table S1. Well construction
Table S2. PFAS abbreviations
Table S3. PFAS physicochemical properties
Table S4. Key to biomarkers
Table S5. Groundwater PFAS extended
Table S6. Groundwater PFAS summary
Table S7. Groundwater PFAS over time Linear Regression
Table S8. Fish plasma PFAS extended
Table S9. Fish plasma PFAS summary
Table S10. Fish plasma PFAS ANOVA for treatment x time
Table S11. Fish plasma CF_B
Table S12. Fish plasma CF_B ANOVA for treatment x time
Table S13. Fish plasma PFAS on day 7 in acclimated fish extended
Table S14. Fish plasma PFAS on day 7 in acclimated fish summary
Table S15. Fish plasma CF_B on day 7 in acclimated fish
Table S16. Fish plasma CF_B on day 7 in acclimated fish ANOVA for treatment x time
Table S17. Mobile lab fish biomarkers extended
Table S18. Mobile lab fish biomarkers summary
Table S19. Testis microarray enriched pathways
Table S20. Testis microarray hallmark signatures
Table S21. Testis microarray biological processes
Table S22. Testis microarray top biological processes
Table S23. Liver microarray enriched pathways
Table S24. Liver microarray biological processes
Table S25. Liver microarray top biological processes
Table S26. Liver microarray upstream signatures FTA1-100% vs. REF
Table S27. Liver microarray upstream signatures FTA2-100% vs. REF
Table S28. Liver microarray upstream signatures FTA2-50% vs. REF

Methods Supporting Information

S1. Study Site and Exposure Waters

The study was conducted at Joint Base Cape Cod (JBCC; Massachusetts, USA) at the U.S. Geological Survey (USGS) Toxic Substances Hydrology Groundwater Research Site that encompasses well-characterized contaminant plumes originating from historical use of aqueous film-forming foam (AFFF) containing per- and polyfluoroalkyl substances at a former fire-training area (FTA).[1-8] The FTA was used from about 1958 to 1985, during which time fuel products and waste organic chemicals, including chlorinated solvents, were burned on open sandy areas or concrete pads; use of AFFF began in about 1970. In 1997, contaminated sandy sediments at the site were excavated to a depth of about 11 m, thermally treated to remove the fuel residues and solvents (but not at temperatures sufficiently high to remove PFAS), and placed back into the excavation. Additional AFFF was used at the site in 1997 to suppress a fire at the treatment facility. Although this site underwent partial remediation to remove fuel constituents and volatile organic compounds,[9-12] elevated PFAS persist in downgradient groundwater, surface water, and biota.[5,6,13]

Sampling wells at the reference (REF) and fire-training area sites (FTA1, FTA2) consisted of 10.2-cm PVC casings with 1.4-m screened intervals (0.25-mm slots). The water table was approximately 15.8 m below land surface at all locations. Screen tops were installed at 17.9 m below land surface at REF, 23.8 m at FTA1, and 16.5 m at FTA2, corresponding to an elevation near 31.2 m above mean sea level at the REF location. Hydrogeologic and well construction details are provided below and in Table S1.

S2. Groundwater PFAS and Co-Occurring Contaminant Analytical Chemistry

Groundwater samples were collected at the point of inflow to the mobile laboratories and analyzed for PFAS using a direct-injection liquid chromatography–tandem mass spectrometry (DI-LC–MS/MS) method with isotope-dilution quantification, following U.S. Geological Survey National Water Quality Laboratory (NWQL) procedures (USGS Method O-4441-22; Laboratory Schedule 9660).[14]

Unfiltered groundwater samples were collected directly into polypropylene centrifuge tubes and amended with methanol to achieve approximately 50% (v/v) organic content to minimize sorptive losses of hydrophobic PFAS. A mixture of isotope-labeled internal standards was added to each sample prior to centrifugation. Samples were mixed, centrifuged to remove particulates, and an aliquot of the centrifuge supernatant was transferred to polypropylene autosampler vials containing an injection internal standard.

Samples were analyzed by liquid chromatography–tandem mass spectrometry operated in negative electrospray ionization mode with dynamic multiple-reaction monitoring. Chromatographic separation was achieved on a C18 column using a binary gradient of ammonium acetate in water and methanol. Target analytes were identified based on retention time, precursor-to-product-ion transitions, and qualifier ion ratios relative to calibration standards. Quantification was performed using isotope-dilution calibration with multi-point calibration curves ($r^2 \geq 0.99$).

Limits of quantification were defined according to USGS NWQL criteria and were compound-specific, matrix-validated, and adjusted for dilution when necessary. Quality assurance and quality control procedures included reagent blanks, laboratory blanks, continuing

calibration verification standards, isotope-dilution standard recovery evaluation, and replicate analyses within each analytical batch.

Target analytes included legacy perfluoroalkyl sulfonates and carboxylates, fluorotelomer sulfonates, sulfonamide precursors, and selected PFAS substitutes commonly associated with aqueous film-forming foam–impacted groundwater. Compound abbreviations and physicochemical characteristics are summarized in Tables S2–S3. Further details on PFAS methods are reported in Vajda et al.[15-17]

To assess non-PFAS constituents, volatile organic compounds were analyzed according to U.S. EPA Method 8260C[18] by RTI Laboratories (Livonia, Michigan). Groundwater collected from REF, FTA1, and FTA2 in December 2023 was stored in 40-mL amber glass vials, chilled, and analyzed upon receipt. This targeted VOC screen was intended to provide limited context on selected co-occurring contaminants and does not represent a comprehensive characterization of all non-PFAS constituents in exposure waters.

S3. Mobile Laboratory Fish Exposure Conditions

Groundwater was pumped continuously from each well through high-density polyethylene tubing at 10 L min^{-1} using stainless-steel submersible pumps (Grundfos RediFlo-3; Geotech Environmental Equipment, Denver, Colorado). Pumped water entered stainless-steel head tanks positioned above the mobile laboratories and flowed by gravity to splitter tanks for temperature equilibration at 20 ± 1 °C prior to distribution to aquaria. Each aquarium received approximately 200 mL min^{-1}, ensuring a complete water replacement roughly every four hours. The mobile-laboratory configuration maintained controlled flow, temperature, lighting, diet, and aeration while preserving the native chemical composition of each groundwater source. All

wetted materials were inert (glass, stainless steel, high-density polyethylene, or polyether ether ketone). Fish experienced a 14 hours light:10 hours dark photoperiod and a standardized diet (Zeigler Finfish Starter #1; Zeigler Brothers, Inc., Gardners, Pennsylvania). Additional system details are reported elsewhere.[8,19]

Fathead minnows (*Pimephales promelas*) were selected because of their extensive use in ecotoxicology and documented sensitivity to PFAS.[20,21] Reproductively recrudescent 12-month-old males were obtained from Aquatic Biosystems (Fort Collins, Colorado) and shipped overnight to the study site. All activities were conducted under approved University of Colorado Denver Institutional Animal Care and use Committee (CU Denver IACUC #00698) and U.S. Geological Survey (USGS/WARC/LFT #2022-0) protocols and followed established fish care guidelines.[22]

Exposure treatments varied by year based on available groundwater sources and study objectives. In 2018, treatments were REF and FTA1 (100%). In 2019, treatments were REF, FTA1 (100%), and FTA1 (50% and 25%). In 2021, treatments were REF, FTA1 (100%), FTA2 (100%), and FTA2 (50%). Intermediate treatments (50% and 25%) were prepared by blending source groundwater with REF at splitter tanks using controlled volumetric inflows from each source to preserve the PFAS mixture composition of the undiluted FTA groundwater. Dilutions were generated hydraulically by calibrating inflow rates from the REF well and the relevant FTA well prior to mixing. For 50% treatments, groundwater from the REF and FTA wells was delivered to the splitter tanks at identical flow rates of 1,200 mL min^{-1} each (1:1). For 25% treatments, groundwater was delivered at 600 mL min^{-1} from the FTA well and 1800 mL min^{-1} from the REF well (1:3). These flow-controlled inflows resulted in proportional volumetric mixing of REF and FTA groundwater under continuous-flow conditions. Nominal PFAS

concentrations in dilution treatments were estimated from measured concentrations in the undiluted FTA groundwater and the known volumetric mixing ratios, rather than measured directly. Flow rates to each splitter tank were verified weekly throughout each exposure period and did not require further adjustment. Twenty-one-day exposures were conducted in 2018, 2019, and 2021. At day-0, a cohort of males was sampled to establish initial controls (IC). The remaining fish were distributed randomly to replicate aquaria for each treatment. Fish were sampled from each treatment on day-7 and day-21. Each sampling consisted of two aquaria each containing five male fish (n=10 per treatment per time point). Survival was evaluated daily throughout the exposure period and mortality was recorded.

In 2019 and 2021, a parallel acclimation design was used to separate PFAS mixture effects from handling or osmotic stress. A subset of fish was maintained in REF groundwater from day-0 through day-13. On day-13, ten randomly selected REF-acclimated fish were sampled as acclimated ICs (two aquaria, five males each). The remaining acclimated fish were randomly assigned to treatments for a seven-day exposure. In 2019, assignments were REF, FTA1-100%, and FTA1-50%. In 2021, assignments were REF, FTA1-100%, FTA2-100%, and FTA2-50%. Sampling at the end of the seven-day acclimated exposures used two aquaria of five males per treatment (n=10).

Unless otherwise noted, aquaria were stocked with five males per aquarium, and fish were randomly assigned to tanks and treatments using a computer-generated randomization list to balance body length and mass distributions among tanks. Tanks assigned to the same treatment were positioned in alternating order to minimize positional bias. Husbandry and sampling personnel followed a scripted sequence to avoid systematic order effects.

S4. Fish Biomarker Analysis

At each sampling point, fish were anesthetized using tricaine methanesulfonate (MS-222; Syndel, Ferndale, Washington) and euthanized prior to dissection.[23] Fork length was measured to the nearest millimeter and body mass to the nearest 0.01 g. Blood was collected from the caudal vein using pre-heparinized microcentrifuge tubes. Secondary sex traits, including nuptial tubercle number and fat pad prominence, were scored using established ordinal criteria.[24] Gonadosomatic index (GSI) and hepatosomatic index (HSI) were calculated from wet tissue masses.[25,26]

Each testis pair was separated and allocated as follows. One testis was reserved for sperm-quality analyses, and the contralateral testis was divided for histology and mRNA analysis as detailed below. In 2018, liver tissue was frozen for vitellogenin mRNA quantification by qPCR. In 2021, testis tissue was frozen for microarray analysis. Plasma was collected whenever sample volume permitted and was analyzed for PFAS during the 2021 experiments. All frozen samples were stored at −80 °C until analysis.

Sperm motility and spermatogenic cell distributions were evaluated in 2019 and 2021. One testis per male was shipped overnight in Ginsburg Fish Ringers buffer (pH 7.37, 290 mOsm kg^{-1}) containing 1% antibiotic/antimycotic solution (Thermo Fisher Scientific, Waltham, Massachusetts) to the U.S. Geological Survey Wetlands and Aquatic Research Center (Lafayette, Louisiana). In 2021, some shipments were delayed up to 48 h; however, only motility data obtained within 24 h of dissection were included in statistical analyses. Upon receipt, small tissue streaks were plated on Luria agar to confirm absence of microbial contamination that could lessen cell quality. Each testis was weighed and divided for computer-assisted sperm motion analysis (CASA) and flow cytometry.

For CASA, cells were activated in tap water and loaded onto 20 μm chambered slides. Motility was recorded with a Zeiss Axio Lab.A1 microscope (Oberkochen, Germany) at 60 frames per second and analyzed with SpermVision software (MOFA Global, Verona, Wisconsin). Standard tracking parameters were used, including a cell identification area of 8–48 μm^2 with minimal particle filtration. Cells were classified as immotile when the average orientation change of the head plus distance straight line (DSL) was less than 25 μm or when DSL was less than 1.0 μm.

For flow cytometry, tissue was gently triturated in GFR buffer, fixed with 4% paraformaldehyde, filtered through 30 μm mesh, and stained with Syto BC nucleic acid dye. Samples were analyzed on a Northern Lights-1000 flow cytometer (Cytek Biosciences, Fremont, California) using SpectroFlo v3.0.3 software (Cytek Biosciences). Percentages of sperm, spermatids, and diploid forms were determined using cytograms and density plots in FlowJo (Tree Star, Ashland, Oregon). Analytical approaches are described in Jenkins et al.[27-30]

Formalin-fixed testes from day-0 and day-21 males (2018, 2019, 2021) were dehydrated through graded ethanol, cleared in Histoclear (Thermo-Fisher Scientific), embedded in Paraplast (Leica Biosystems, Minneapolis, Minnesota), sectioned at 5 μm, and stained with hematoxylin and eosin.[26,31] Histological evaluation included sperm density and abundance, spermatogenetic tubule lumen shape, sperm presence in collecting ducts, germinal epithelial stage, testis stage, granulomatous inflammation, interstitial proteinaceous fluid, interstitial fibrosis, and testis-ova occurrence.[32,33] Criteria and ordinal scoring are provided in Table S4.

For cell-cycle and apoptosis biomarkers, day-0 and day-21 sections from 2018 and 2019 were analyzed using PCNA immunostaining and TUNEL assays following D'Andrea et al.[34]

Ten serial sections per fish were scored to quantify PCNA- and TUNEL-positive staining in germinal epithelium and luminal cells. Scoring approaches are presented in Table S4.

Frozen liver fragments from 2018 REF and FTA1 fish were analyzed by quantitative real-time PCR for vitellogenin mRNA. Total RNA was extracted using the RNeasy Plus Mini Kit (Qiagen, Germantown, Maryland) according to the manufacturer's protocol. RNA integrity was confirmed using an Agilent 2100 BioAnalyzer (Agilent Technologies, Santa Clara, California) and RNA 6000 Nano Kits (Agilent Technologies). Only samples with RIN greater than 6 were used. RNA concentrations and purity (260/280 and 260/230 ratios) were assessed using a NanoDrop-1000 spectrophotometer (Thermo Fisher Scientific). qPCR was performed on a StepOnePlus real-time PCR system (Applied Biosystems, Foster City, California) with the Power SYBR Green RNA-to-Ct 1-Step Kit (Applied Biosystems). Vitellogenin mRNA expression was normalized to 18S rRNA using QuantumRNA universal primers (Thermo Fisher, Carlsbad, California). Primer sequences follow Biales et al.[35] Melting-curve analysis verified amplification specificity. Relative expression values were calculated from cycle threshold data using StepOnePlus software (Thermo Fisher, Carlsbad, CA).

Testis and liver fragments were collected from 2021 males that were acclimated for thirteen days in REF water and then exposed for seven days to REF, FTA2-100% or FTA2-50%. Total RNA was extracted using TRIzol reagent (Thermo Fisher Scientific, Waltham, MA), and RNA quality and integrity were verified with an Agilent 2100 BioAnalyzer using the RNA 6000 Nano assay. Samples with RIN values greater than 6 were purified using the RNeasy Mini Kit (Qiagen, Germantown, MD) prior to labeling. Testes from FTA1-100% fish were excluded from microarray analysis due to low RNA recovery. Transcriptomic profiling used a fathead minnow-specific 8 × 60K custom microarray platform (GPL15775; Agilent Technologies, Carlsbad, CA)

described in Garcia-Reyero et al.[36] Labeling and hybridization were carried out using the One-Color Microarray-Based Gene Expression Analysis Low Input Quick Amp Labeling Kit (Agilent v6.5, May 2010) with 100 ng RNA per sample. Arrays were scanned at 3 μm resolution on an Agilent G2505B Microarray Scanner, and raw data were extracted with Feature Extraction Software v9.5 (Agilent Technologies, Santa Clara, CA).

Transcriptomic data were quality-filtered, $\log_2$-transformed, and quantile-normalized prior to analysis in JMP Genomics v9.0 (SAS Institute, Cary, North Carolina). One-way ANOVA with Benjamini–Hochberg false discovery rate correction ($q \leq 0.05$) was used to identify differentially expressed genes.[37] Targeted qPCR validation of selected transcripts was not conducted; therefore, transcriptomic interpretation emphasizes statistical filtering and pathway-level concordance. No transcripts passed FDR correction in testes; therefore, genes with unadjusted $p < 0.05$ were retained for exploratory interpretation.[38] Several transcripts passed FDR correction in the liver, and these are reported in the results. Pathway enrichment analyses were conducted using iPathway software (Advaita Corporation, Plymouth, Michigan) to identify biological processes and hallmark signatures based on KEGG and Gene Ontology resources.[39-41] The complete microarray dataset for testis and liver is archived in the NCBI Gene Expression Omnibus under accession GSE261668.

Liver differentially expressed genes from each treatment comparison were analyzed in iPathwayGuide (Advaita Corporation, Ann Arbor, MI) using curated chemical, drug, and toxicant interactions in the Advaita Knowledge Base (AKB v18.1) to identify predicted upstream signatures associated with the observed transcriptomic responses. The analysis integrates enrichment of differentially expressed genes with directional concordance between observed expression changes and curated signed regulator-gene interactions and combines these lines of

evidence to rank predicted upstream chemicals, drugs, and toxicants. Predicted signatures were interpreted as transcriptional response analogs rather than as evidence that the named compounds were present in exposure water. Because the reference signatures are not liver-specific, inferred responses may also reflect profiles derived from other tissues or systems. [42,43]

S5. Plasma PFAS Extraction and Analysis: Biotic Concentration Factors

Plasma collected in 2021 was analyzed for PFAS at the U.S. Geological Survey Eastern Ecological Science Center (Kearneysville, West Virginia). Because of limited sample volumes (mean sample volume 8.5 ± 2.4 µL), a modified small-volume serum extraction protocol was used. Plasma samples were prepared for analysis by first thawing and vortexing. An aliquot of plasma, ranging from 2-10 µL depending on the sample, was transferred to a 2 mL polypropylene centrifuge tube. To normalize the extracted volume across all samples, the aliquot of plasma was brought to a total volume of 20 µL with LC-MS grade water. A methanolic dosing stock containing extraction internal standard (0.02-0.024 ng μL^{-1}) was vortexed, and 10 µL was added to the centrifuge tube. Next, 40 µL of a 0.1% formic acid solution in LC-grade water was added to the centrifuge tube. The sample was vortexed for at least 10 seconds. Following this, 180 µL of cold (-20°C) acetonitrile was added to the centrifuge tube and vortexed for at least 10 seconds. The centrifuge tube containing the extract was centrifuged at 12,500 x g for 5 minutes at room temperature (approximately 25°C). To a fresh polypropylene LC vial, 140 µL of LC-grade water was added. Subsequently, 200 µL of the extracted plasma supernatant was added to the LC vial followed by 50 µL of LC-grade methanol. A methanolic dosing stock containing injection internal standards (0.005-0.02 ng/µL) was vortexed, and 10 µL was dosed. The LC vial

was capped and vortexed for analysis. For sample plasma concentrations that fell above the calibration curve range, dilutions were performed according to the following approach. To a fresh polypropylene LC vial, 140 µL of LC-grade water was added. Next, 20 µL of the extracted plasma supernatant was added to the LC vial. The extract was further diluted by adding 180 µL of extracted water (water extracted using the same approach as samples). Following this, 50 µL of LC-grade methanol was added to the centrifuge tube. A methanolic dosing stock containing injection internal standards (0.005-0.02 ng/µL) was vortexed, and 10 µL was dosed. The LC vial was capped and vortexed for analysis.

Analysis was completed on a Vanquish™ Flex liquid chromatograph (Thermo Fisher Scientific, Waltham, MA) coupled to a Thermo Scientific™ Orbitrap Exploris™ 120 high resolution mass spectrometer operated in negative polarity. A Thermo Scientific™ TriPlus™ RSH EQuan 850 System (Thermo Fisher Scientific) outfitted with polyether ether ketone tubing was used for sample injection of 80 µL into a 100 µL sample loop, and an Agilent InfinityLab Poroshell HPH-C18 guard column (Agilent Technologies) was installed inline to prevent contamination from the mobile phase. A Waters™ Atlantis™ Premier BEH C18 AX analytical column (Waters Corporation, Milford, Massachusetts), maintained at 40 degrees Celsius, was used for compound separation. The eluent flow rate was 0.5 milliliters per minute and consisted of mobile phase A (2 millimolar ammonium acetate in LC-grade water) and B (LC-grade methanol). The run started with 90% A for 1.0 minute before decreasing to 45% A over 0.9 minutes. At 1.9 minutes the eluent composition was further decreased from 45% A to 2% A over 8.6 minutes and held until 14.5 minutes. From 14.5 to 14.6 minutes the eluent composition was ramped back to the initial composition of 90% A and held until 19 minutes. Data acquisition was

conducted using full scan MS with data-dependent MS/MS under negative ion mode using electrospray ionization.

Quantitation was based on isotope dilution and multi-point calibration curves (%RSD ≤ 20%). The method detection limit (MDL) for each analyte was determined according to U.S. Environmental Protection Agency Appendix B to Part 136, Title 40 "Definition and Procedure for the Determination of the Method Detection Limit - Revision 2".[44] Compound specific MDL for plasma were adjusted based on the sample dilution factor. The minimum reporting limit (MRL) was determined by multiplying the MDL by a factor of 3.18. QA/QC included instrument blanks, method blanks, ongoing precision recovery samples, matrix spikes, and isotope-labeled surrogate recoveries.[17] Plasma biotic concentration factors (CF_B) were calculated as the ratio of plasma PFAS concentration to the mean groundwater concentration for each analyte.

S6. Statistical Analyses

Temporal trends in groundwater ∑PFAS were evaluated by simple linear regression at each site-year, and slope, intercept, R^2, and p-values are reported. Plasma PFAS concentrations and CF_B were analyzed using two-way ANOVA with treatment and time as fixed factors and a treatment by time interaction. Significant effects at $p < 0.05$ were followed by Tukey post-hoc tests. Organismal indices and secondary sex characteristics were tested for normality (Shapiro–Wilk) and log-transformed when necessary; one-way ANOVA with Tukey post-hoc tests or Kruskal–Wallis with Dunn's post-hoc tests for ordinal scores was used as appropriate. Analyses were performed in GraphPad Prism v10.5 (GraphPad Software, Boston, MA). Detailed statistical results are presented in the SI tables (Tables S7, S10, S12, S16).

S7. Results

Full groundwater PFAS results for 2018, 2019, and 2021 are reported in in Tables S5-S7 and Vajda et al.[15-17] Full fathead minnow sperm quality analysis are reported in Jenkins et al.[28,29] Fish plasma PFAS results are reported in Tables S8-S16. Fish biomarker responses are reported in Tables S17-S28.

Any use of trade, firm, or product names is for descriptive purposes only and does not imply endorsement by the U.S. Government.

LITERATURE CITED

1. LeBlanc, D. R. Sewage plume in a sand and gravel aquifer, Cape Cod, Massachusetts. U.S. Geological Survey Water-Supply Paper 2218; U.S. Geological Survey: Reston, VA. **1984**, 28 p**.** http://doi.org/10.3133/wsp2218
2. Barber, L. B., II; Thurman, E. M.; Schroeder, M. P.; LeBlanc, D. R. Long-term fate of organic micropollutants in sewage-contaminated ground water. *Environ. Sci. Technol*. **1988**, *22*, 205-211. https://doi.org/10.1021/es00167a012
3. Field, J. A.; Leenheer, J. A.; Thorn, K. A.; Barber, L. B.; Rostad, C.; Macalady, D. L.; Daniel, S. R. Identification of persistent anionic surfactant-derived chemicals in sewage effluent and groundwater. *J. Contam. Hydrol.* **1992**, *9*, 55-78. https://doi.org/10.1016/0169-7722(92)90050-O
4. Barber, L. B.; Keefe, S. H.; LeBlanc, D. R.; Bradley, P. M.; Chapelle, F. H.; Meyer, M. T.; Loftin, K. A.; Kolpin, D. W.; Rubio, F. Fate of sulfamethoxazole, 4-nonylphenol, and 17β-estradiol in groundwater contaminated by wastewater treatment plant effluent. *Environ. Sci. Technol*. **2009**, *43*, 4843-4850. https://doi.org/10.1021/es803292v
5. Weber, A. K.; Barber, L. B.; LeBlanc, D. R.; Sunderland, E. M.; Vecitis, C. D., 2017. Geochemical and hydrologic factors controlling subsurface transport of poly- and perfluoroalkyl substances, Cape Cod, Massachusetts. *Environ. Sci. Technol*. **2017**, *51*, 4269–4279. https://doi.org/10.1021/acs.est.6b05573
6. Tokranov, A. K.; LeBlanc, D. R.; Pickard, H. M.; Ruyle, B. J.; Barber, L. B.; Hull, R. B.; Sunderland, E. M.; Vecitis, C. D. Surface-water/groundwater boundary effects on seasonal PFAS concentrations and PFAA precursor transformations. *Environ. Sci. Process. Impact.* **2021**, *23*, 1893-1905. https://doi.org/10.1039/D1EM00329A
7. Tokranov, A. K.; Pickard, H. M.; LeBlanc, D. R.; Ruyle, B. J.; Hull, R. B.; Barber, L. B.; Repert, D. A.; Sunderland, E. M.; Vecitis, C. T. Concentrations of per- and polyfluoroalkyl substances (PFAS) and related chemical and physical data at and near surface-water/groundwater boundaries on Cape Cod, Massachusetts, 2016-19. U.S. Geological Survey data release https://doi.org/10.5066/P9HPBFRT: U.S. Geological Survey; Washington, DC, **2021**.]
8. Barber, L. B.; Pickard, H. M.; Alvarez, D. A.; Becanova, J.; Keefe, S. H.; LeBlanc, D. R.; Lohmann, R.; Steevens, J. A.; Vajda, A. M. Uptake of per- and polyfluoroalkyl substances by fish, mussel, and passive samplers in mobile-laboratory exposures using groundwater from a contamination plume at a historical fire training area. *Environ. Sci. Technol.* **2023**, *57*, 5544-5557. https://doi.org/10.1021/acs.est.2c06500
9. Air Force Civil Engineer Center (AFCEC). Soil thermal treatment program remedial action summary report, AFCEC/MMR Installation Restoration Program, AFCEC Administrative Record document no.131868, **1999**. [Accessed January 4, 2026, at https://ar.cce.af.mil/]
10. Air Force Civil Engineer Center (AFCEC). Final closure report, FTA-1 Site. AFCEC/MMR Installation Restoration Program, AFCEC Administrative Record document no.131431, **2000**. [Accessed January 4, 2026, at https://ar.cce.af.mil/]
11. Air Force Civil Engineer Center (AFCEC). Final supplemental remedial investigation/feasibility study work plan for 1,4-dioxane and perfluorinated compounds at Ashumet Valley, Joint Base Cape Cod, MA. AFCEC/JBCC Installation Restoration

Program, AFCEC Administrative Record document no. 472031, **2016**. [Accessed on January 4, 2026, at https://ar.cce.af.mil/]
12. Air Force Civil Engineer Center (AFCEC). Draft Engineering Evaluation/Cost Analysis for Fire Training Area FT-055, Joint Base Cape Cod, Massachusetts, **2022**. [Accessed January 4, 2026, at https://ar.cce.af.mil/]
13. Walsh, H. L.; Blazer, V. S.; Iwanowicz, L. R.; Leblanc, D. R.; Foreman, W. T.; Smith, G.; Brightbill, R. A.; Alvarez, D. A.; Iwanowicz, D. D. Occurrence and tissue distribution of per- and polyfluoroalkyl substances (PFAS) in fishes from waterbodies with point and non-point source contamination. *Aquat. Toxicol.* **2025**, *287*, 107499. https://doi.org/10.1007/s11356-024-35097-6
14. Gray, J.; Kanagy, L.; Kanagy, C.; Anderson, C. A., Determination of per- and polyfluoroalkyl substances in water by direct injection of matrix-modified centrifuge supernatant and liquid chromatography/tandem mass spectrometry with isotope dilution. U.S. Geological Survey Techniques and Methods 5-B13: U.S. Geological Survey; Washington, DC, **2025**, 121 p. https://doi.org/10.3133/tm5B13.
15. Vajda, A. M.; Barber, L. B.; Pickard, H. M.; Alvarez, D. A.; Becanova, J.; Bertolatus, D. W.; Jasmann, J. R.; Keefe, S. H.; LeBlanc, D. R.; Lohmann, R.; Steevens, J. Uptake of per- and polyfluorinated alkyl substances by fathead minnows, freshwater mussels, and passive samplers from contaminated groundwater at a fire-training area, Cape Cod, Massachusetts – chemical and biological data from August to September 2018. U.S. Geological Survey data release, **2023**. https://doi.org/10.5066/P9LCN0EF
16. Vajda, A. M.; Hill, N. I.; Barber, L. B.; Lohmann, R.; Becanova, J.; Vojta, S.; Pickard, H. M.; Bertolatus, D. W.; LeBlanc, D. R. Tissue-specific bioconcentration of per- and polyfluoroalkyl substances by fathead minnows from contaminated groundwater at a fire-training area, Cape Cod, Massachusetts, during 2019 mobile laboratory experiments. U.S. Geological Survey data release, **2024**. http://doi.org/10.5066/P9TGHQWB
17. Vajda, A. M.; Barber, L. B.; Jenkins, J. A.; LeBlanc, D. R.; Lind, H. G.; Martyniuk, C. J.; Dethloff, A. R.; Tokranov, A. K.; Hopkins, Z. R. Lohmann, R. Reproductive biomarker responses in male fathead minnow exposed to per- and polyfluoroalkyl substances contaminated groundwater at a fire-training area, Cape Cod, Massachusetts from 2019 and 2021. U.S. Geological Survey Data Release, **2026**. https://doi.org/10.5066/P13UB8SY
18. U.S. Environmental Protection Agency. Method 8260D (SW-846): Volatile Organic Compounds by Gas Chromatography/Mass Spectrometry (GC/MS), Revision 3. U.S. Environmental Protection Agency: Washington, DC; **2006**.
19. Hill, N. I.; Becanova, J.; Voijta, S.; Barber, L. B.; LeBlanc, D.; Vajda, A. M.; Pickard, H. M.; Lohmann, R. Bioconcentration of per- and polyfluoroalkyl substances and precursors in fathead minnow tissues environmentally exposed to aqueous film-forming foam-contaminated waters. *Environ. Toxicol. Chem.* **2024**, *43*,1795-1806. https://doi.org/10.1002/etc.5926
20. Ankley, G. T.; Kuehl, D. W.; Kahl, M. D.; Jensen, K. M.; Linnum, A.; Leino, R. L.; Villeneuve, D. A. Reproductive and developmental toxicity and bioconcentration of perfluorooctanesulfonate in a partial life-cycle test with the fathead minnow (*Pimephales promelas*). *Environ. Toxicol. Chem.* **2005**, *24*, 2316-2324. https://doi.org/10.1897/04-634R.1

21. Lee, J. J.; Schultz, I. R. Sex differences in the uptake and deposition of perfluorooctanoic acid in fathead minnows after oral dosing. *Environ. Sci. Technol*. **2010,** *44*, 491-496. https://doi.org/10.1021/es901838y
22. American Institute of Fishery Research Biologists, and American Society of Ichthyologists and Herpetologists. *Guidelines for the Use of Fishes in Research*. American Fisheries Society, Bethesda, MD, **2014**, 90 p.
23. Palić, D.; Stoskopf, M. K.; Small, B. C. Anesthetic efficacy of tricaine methanesulfonate, metomidate, and eugenol: effects on plasma cortisol concentration and neutrophil function in fathead minnows (*Pimephales promelas*). *Aquaculture* **2006**, *254*, 675–685. https://doi.org/10.1016/j.aquaculture.2005.11.004
24. Smith, R. J. Effects of 17 Alpha-methyltestosterone on the dorsal pad and tubercles of Fathead minnows (*Pimephales promelas*). *Can. J. Zool.* **1974**, *52*, 1031–1038. https://doi.org/10.1139/z74-137
25. Strange, R. J. Field examination of fishes. In Murphy, B. R.; Willis, D. W., Eds.), Fisheries Techniques (2nd Ed.), American Fisheries Society, Bethesda, MD, **1996**, 433–446.
26. Vajda, A. M.; Barber, L. B.; Gray, J. L.; Lopez, E. M.; Bolden, A. M.; Schoenfuss, H. L.; Norris, D. O. Demasculinization of male fish by wastewater treatment plant effluent. *Aquat. Toxicol*. **2011,** *103*, 213-221. https://doi.org/10.1016/j.aquatox.2011.02.007
27. Jenkins, J. A.; Olivier, H. M.; Draugelis-Dale, R. O.; Eilts, B. E.; Torres, L.; Patiño, R.; Nilsen, E.; Goodbred, S. L. Assessing reproductive and endocrine parameters in male largescale suckers (*Catostomus macrocheilus*) along a contaminant gradient in the lower Columbia River, USA. *Sci. Tot. Environ.* **2014**, *484*, 365-378. https://doi.org/10.1016/j.scitotenv.2013.09.097
28. Jenkins, J. A.; Draugelis-Dale, R. O.; Pinkney, A. E.; Iwanowicz, L. R.; Blazer, V. S. Flow cytometric method for measuring chromatin fragmentation in fixed sperm from yellow perch (*Perca flavescens*). *Theriogenology* **2015**, *83***,** 920-931. https://doi.org/10.1016/j.theriogenology.2014.11.028
29. Jenkins, J. A.; Baudoin, B. A.; Johnson, D.; Barber, L. B.; Vajda, A. M. Assessment of sperm quality parameters from fathead minnows exposed to polyfluorinated alkyl substances (PFAS) at Cape Cod, MA in 2021. U.S. Geological Survey data release, **2026**. https://doi.org/10.5066/P91CBN7X
30. Jenkins, J. A.; Duhon, L. O.; Barber, L. B.; Vajda, A. M. Assessment of sperm quality parameters from fathead minnows exposed to polyfluorinated alkyl substances (PFAS) at Cape Cod, MA in 2019: U.S. Geological Survey data release, **2026**. https://doi.org/10.5066/P93N1AMQ
31. Presnell, J. K.; Schreibman, M. P. *Humason's Animal Tissue Techniques*. Johns Hopkins University Press, Baltimore, MD, **1997**, 572 p.
32. Blazer, V. S. Histopathological assessment of gonadal tissue in wild fishes. *Fish Physiol. Biochem.* **2002**, *26*, 85–101. https://doi.org/10.1023/A:1023332216713
33. Organization for Economic Co-operation and Development (OECD). Guidance Document on the Diagnosis of Endocrine-related Histopathology in Fish Gonads (OECD Series on Testing and Assessment No. 123). OECD Publishing, Paris. **2010.** https://www.oecd.org/content/dam/oecd/en/publications/reports/2010/05/guidance-document-on-the-diagnosis-of-endocrine-related-histopathology-in-fish-gonads_0f8fc8c2/8f7cf3b5-en.pdf

34. D'Andrea, M. R.; Alicknavitch, M.; Nagele, R. G.; Damiano, B. P. Simultaneous PCNA and TUNEL labeling for testicular toxicity evaluation suggests that detection of apoptosis may be more sensitive than proliferation. *Biotech. Histochem.* **2010**, *85*, 195–204. https://doi.org/10.3109/10520290903547778
35. Biales, A. D.; Bencic, D. C.; Flick, R. W.; Lazorchak, J.; Lattier, D. L. Quantification and associated variability of induced vitellogenin gene transcripts in fathead minnow (*Pimephales promelas*) by quantitative real-time polymerase chain reaction assay. *Environ. Toxicol. Chem.* **2007**, *26*, 287–296. https://doi.org/10.1897/06-213R.1
36. Garcia-Reyero, N.; Kennedy, A. J.; Escalon, B. L.; Habib, T.; Laird, J. G.; Rawat, A.; Wiseman, S.; Hecker, M.; Denslow, N.; Steevens, J. A.; Perkins, E. J. Differential effects and potential adverse outcomes of ionic silver and silver nanoparticles in vivo and in vitro. *Environ. Sci. Technol.* **2014**, *48*, 4546–4555. https://doi.org/10.1021/es4042258
37. Benjamini Y.; Hochberg Y. Controlling the false discovery rate: A practical and powerful approach to multiple testing. *J. Roy. Stat. Soc. Ser. B*. **1995**, *57*, 289–300. https://doi.org/10.1111/j.2517-6161.1995.tb02031.x
38. Owzar, K.; Barry, W. T.; Jung, S. -H. Statistical considerations for analysis of microarray experiments. *Clin. Transl. Sci.* **2011**, *4*, 466-477. https://doi.org/10.1111/j.1752-8062.2011.00309.x
39. Ashburner, M.; Ball, C. A.; Blake, J. A.; Botstein, D.; Butler, H.; Cherry, J. M.; Davis, A. P.; Dolinski, K.; Dwight, S. S.; Eppig, J. T.; Harris, M. A.; Hill, D. P.; Issel-Tarver, L.; Kasarskis, A.; Lewis, S.; Matese, J. C.; Richardson, J. E.; Ringwald, M.; Rubin, G. M.; Sherlock, G. The Gene Ontology Consortium. Gene ontology: Tool for the unification of biology. *Nature Gen.* **2000**, *25*, 25–29. https://doi.org/10.1038/75556
40. Gene Ontology Consortium. Creating the Gene Ontology Resource: Design and implementation. *Genome Res.* **2001**, *11*, 1425–1433. http://doi.org/10.1101/gr.180801
41. Kanehisa, M.; Goto, S.; Kawashima, S.; Nakaya, A. The KEGG databases at GenomeNet. *Nucleic Acids Res.* **2002**, *30*, 42–46. https://doi.org/10.1093/nar/30.1.42
42. Draghici, S.; Khatri, P.; Tarca, A. L.; Amin, K.; Done, A.; Voichita, C.; Georgescu, C.; Romero, R. A systems biology approach for pathway level analysis. *Genome Res.* **2007,** *17*, 1537-1545. https://doi.org/10.1101/gr.6202607
43. Donato, M.; Xu, Z.; Tomoiaga, A.; Granneman, J. G.; Mackenzie, R. G.; Bao, R.; Than, N. G.; Westfall, P. H.; Romero, R.; Draghici, S. Analysis and correction of crosstalk effects in pathway analysis. *Genome Res.* **2013,** *23*, 1885-1893. https://doi.org/10.1101/gr.153551.112
44. U.S. Environmental Protection Agency. Appendix B to Part 136—Definition and Procedure for the Determination of the Method Detection Limit—Revision 2. *Electronic Code of Federal Regulations (eCFR)*, Title 40, Part 136, EPA 821-R-16-006. **2017.** https://www.federalregister.gov/documents/2017/08/28/2017-17271/clean-water-act-methods-update-rule-for-the-analysis-of-effluent

Figure S1. Summary of organismal endpoints across treatments.

Heat map of standardized (z-score) organismal responses in fathead minnows (*Pimephales promelas*) exposed to PFAS-contaminated groundwater across study years (2018, 2019, 2021). Endpoints include mortality, secondary sex characteristics, sperm motility, and histopathology measured on exposure day-21 for Reference (REF), Fire-training area site-1 (FTA1), and Fire-training area site-2 (FTA2) treatments (n = 6–10 per treatment). Blank cells indicate endpoints not assessed. Greater impairment occurred in FTA2 mixtures relative to PFOS-dominated FTA1 mixtures despite overlapping total PFAS concentrations (∑PFAS) in some treatments, consistent with mixture-dependent biological responses.

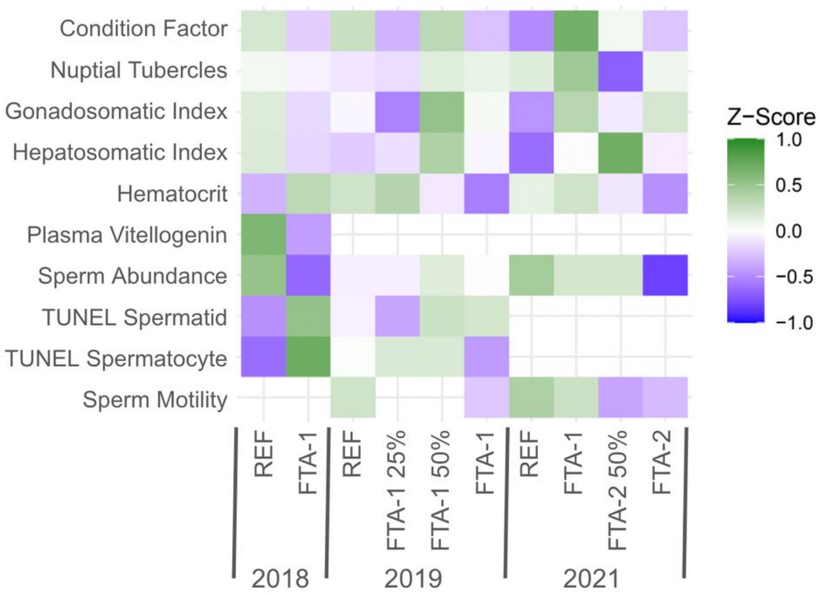

Figure S2. PFOA, PFHxS and PFOS water concentrations across treatments. Groundwater concentrations (μg L^{-1}) of PFOA, PFHxS, and PFOS during 2021 exposures for Reference (REF), Fire-training area site-1 (FTA1), and Fire-training area site-2 (FTA2) treatments. Concentrations for FTA2-50% were estimated as 50% of measured FTA2-100% values. Shaded bands represent 95% confidence intervals. See Table S2 for individual PFAS abbreviations.

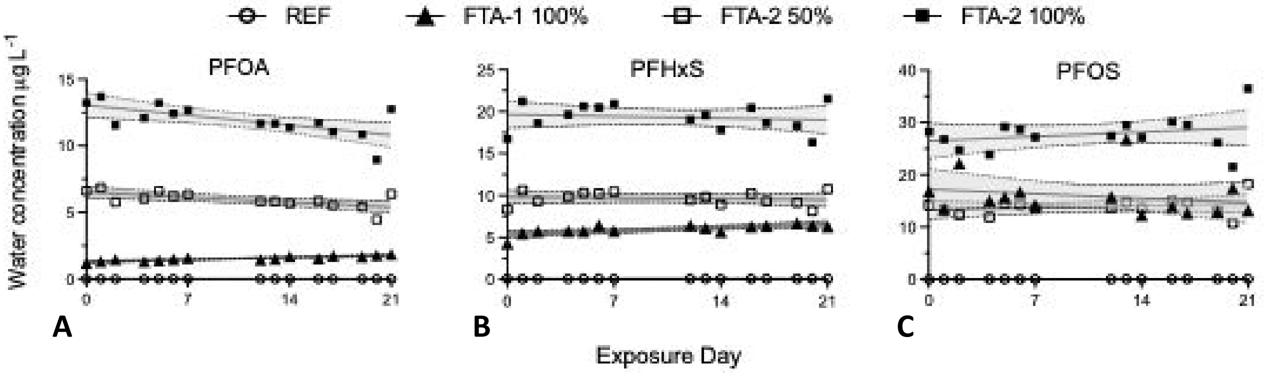

Figure S3. Temporal changes in ∑PFAS in water. Total groundwater PFAS concentrations (∑PFAS**;** µg L^{-1}) during exposures in (A) 2018, (B) 2019, and (C) 2021 for Reference (REF), Fire-training area site-1 (FTA1), and Fire-training area site-2 (FTA2) treatments Shaded bands indicate 95% confidence intervals.

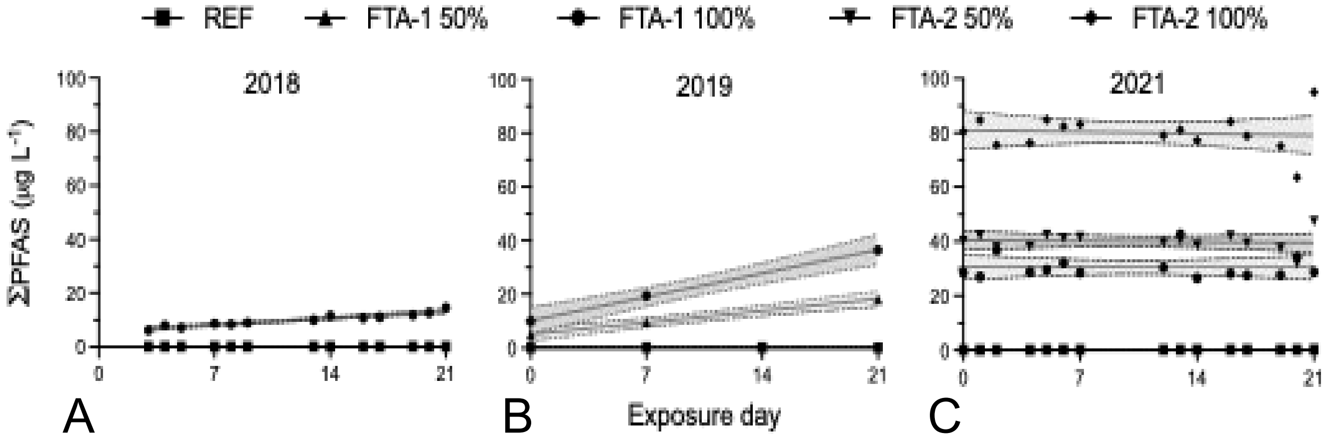

Figure S4. Plasma ∑PFAS temporal dynamics. Plasma ∑PFAS concentrations (ng mL^{-1}) in fathead minnows (*Pimephales promelas*) exposed to groundwater in 2021 for Reference (REF), Fire-training area site-1 (FTA1), and Fire-training area site-2 (FTA2) treatments.

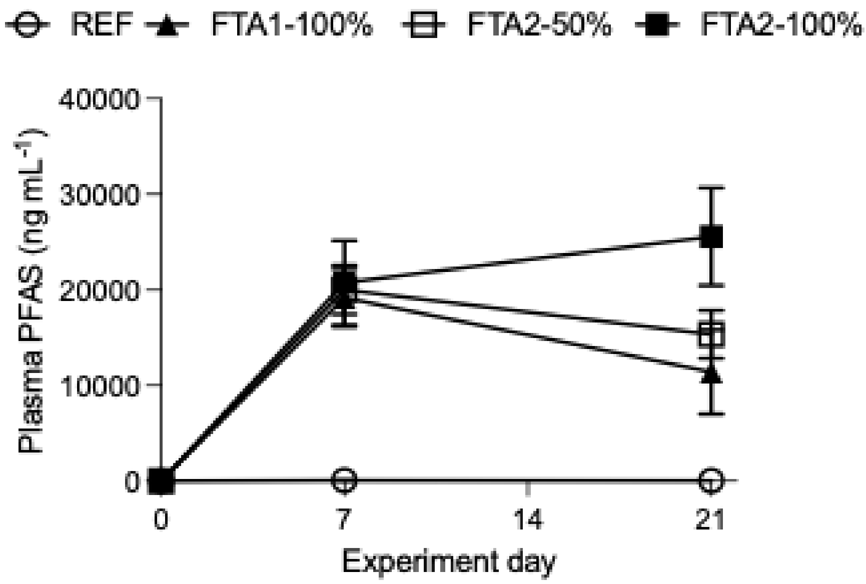

Figure S5. **Plasma PFAS mixture composition**. (A) Fathead minnow (*Pimephales promelas*) plasma PFAS concentrations (ng mL⁻¹) and (B) proportional PFAS composition during 2021 exposures to Reference (REF), Fire-training area site-1 (FTA1), and Fire-training area site-2 (FTA2) treatments. See Table S2 for individual PFAS abbreviations.

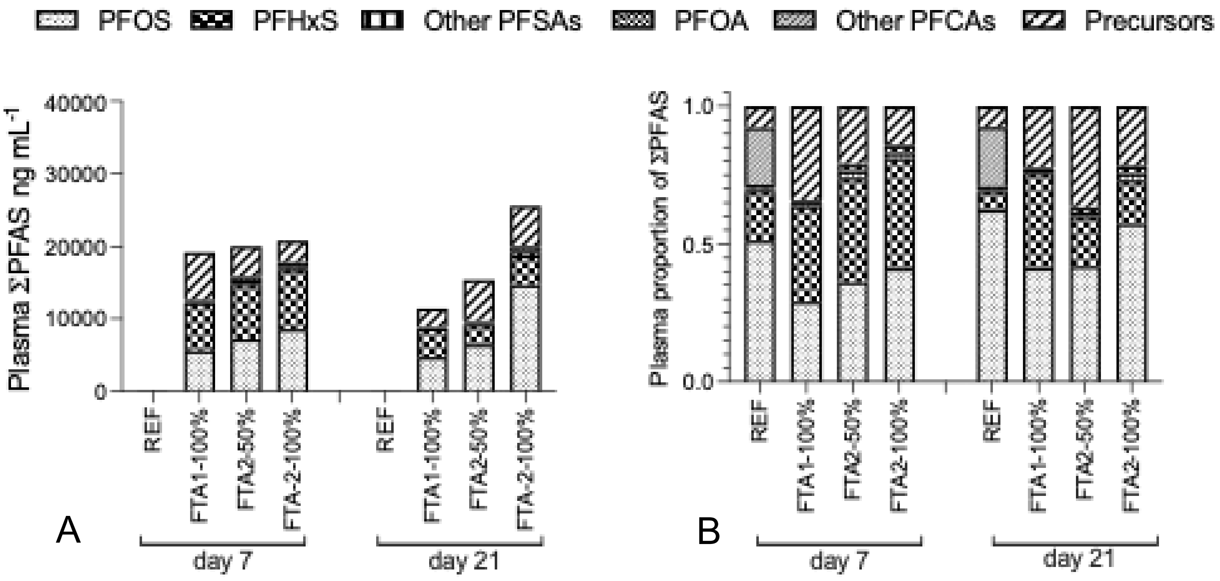

Figure S6. **Plasma concentration of individual PFAS**. Plasma PFAS concentration (ng mL^{-1}) in fathead minnow (*Pimephales promelas*) from 2021 day-0 initial control, day-7 and day-21 for Reference (REF), Fire-training area site-1 (FTA1), and Fire-training area site-2 (FTA2) treatments. Annotation indicates significant (p<0.05) main- and interaction-effects from 2-way ANOVA (Tables S8-S9) (significant effects of Treatment (T), Time (Ti), Interaction (I). See Table S2 for individual PFAS abbreviations.

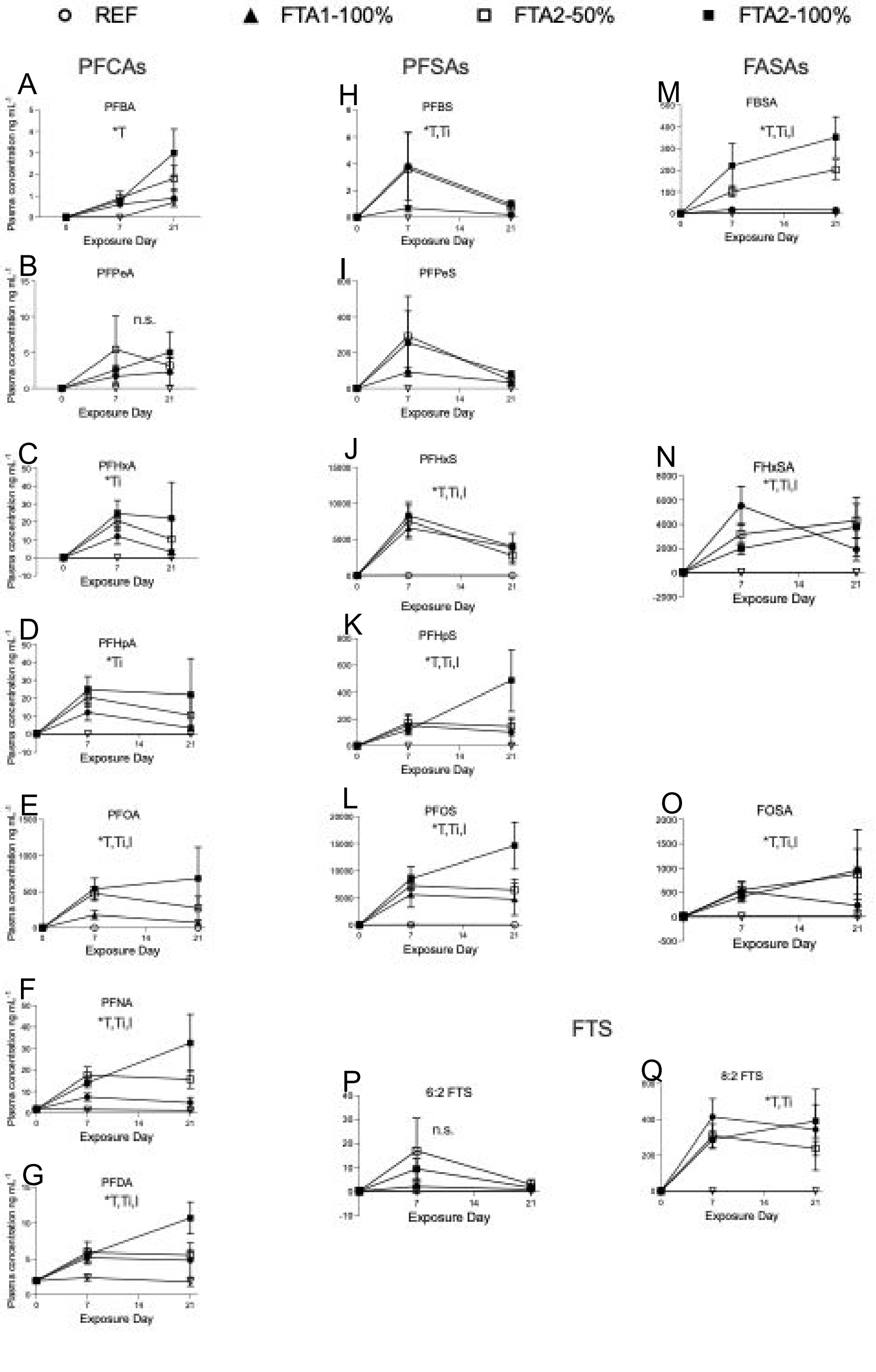

Figure S7. Plasma PFAS biotic concentration factors (CF_B; dimensionless) for perfluoroalkyl carboxylic acids (PFCA), perfluoroalkyl sulfonic acids (PFSA), fluorotelomer sulfonic acids (FTS) and perfluoroalkyl sulfonamides (FASA) detected in plasma of male fathead minnow (*Pimephales promelas*) on exposure day-7 (A) and exposure day-21 (B) for Reference (REF), Fire-training area site-1 (FTA1), and Fire-training area site-2 (FTA2) treatments. See Table S2 for individual PFAS abbreviations.

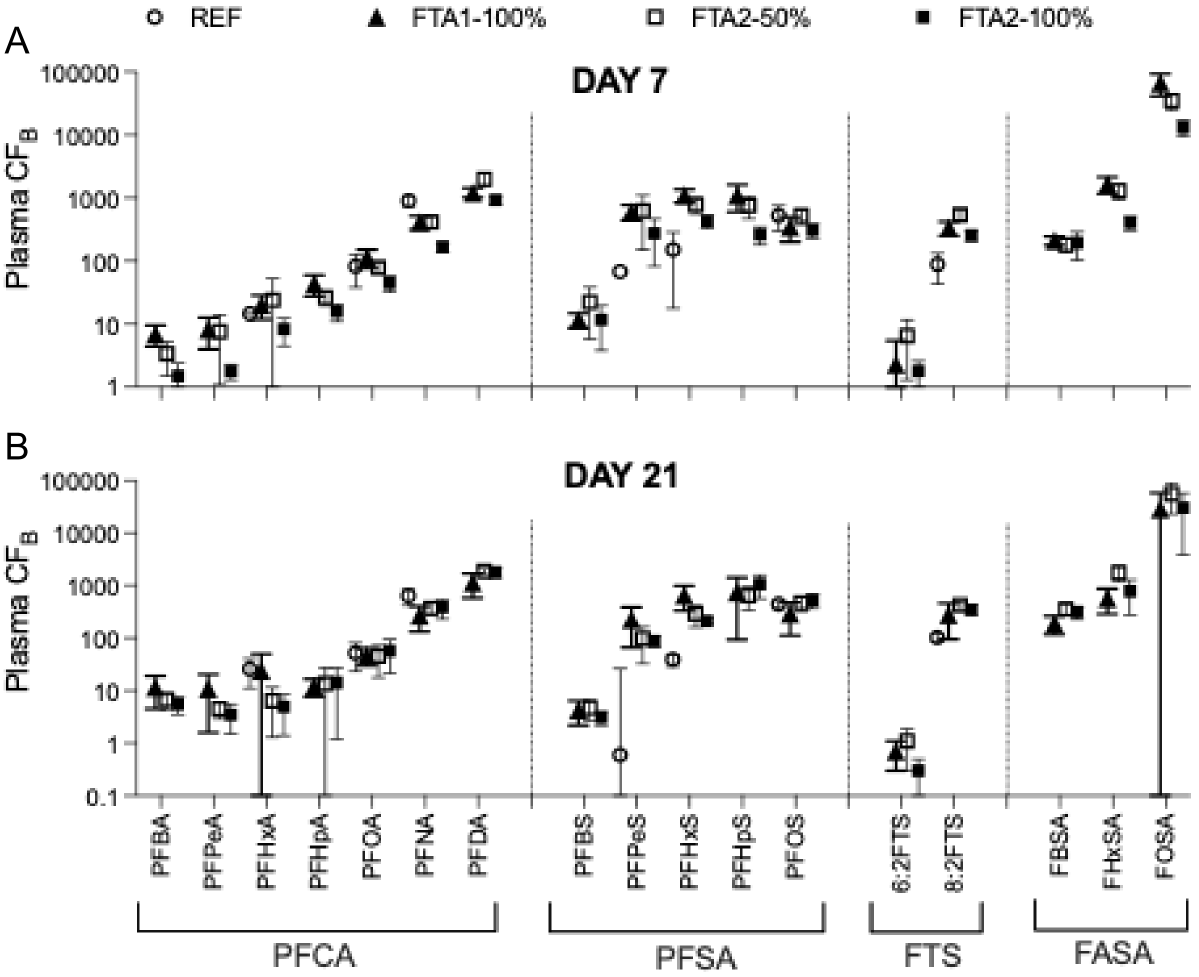

Figure S8. Plasma PFAS biotic concentration factors (CF_B; dimensionless) for PFAS detected in plasma of fathead minnow (*Pimephales promelas*) from 2021 on exposure day-7 and exposure day-21 for Reference (REF), Fire-training area site-1 (FTA1), and Fire-training area site-2 (FTA2) treatments. [Annotation indicates significant ($p<0.05$) main- and interaction-effects from 2-way ANOVA (see Table S10); [significant effects of Treatment (T), Time (Ti), Interaction (I); see Table S2 for individual PFAS abbreviations].

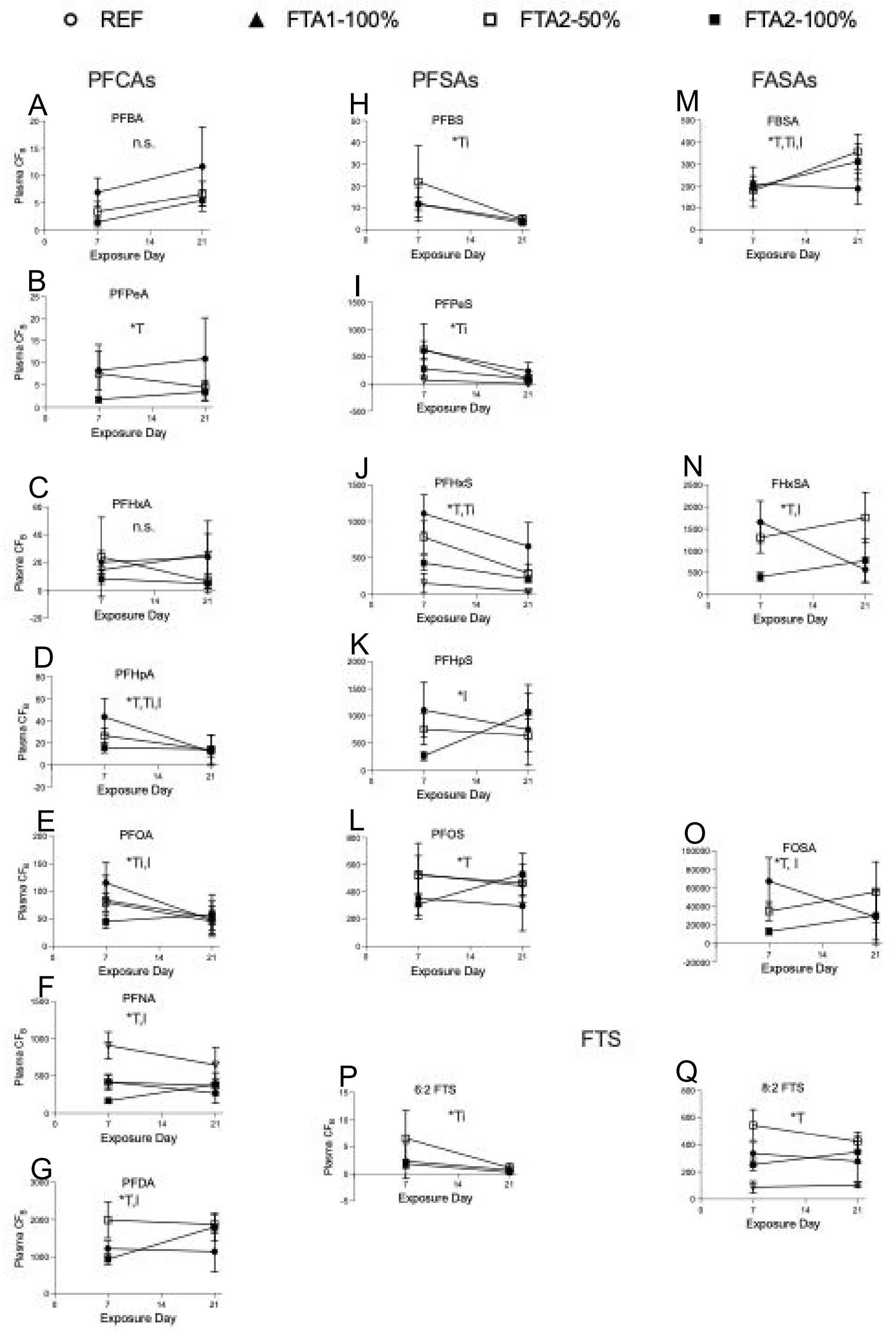

Figure S9. Plasma concentration (ng mL^{-1}) and composition of PFOS, PFHxS, other perfluoroalkyl carboxylic acids (PFCA), other perfluoroalkyl sulfonic acids (PFSA), and PFAS precursors in fathead minnow (*Pimephales promelas*) on exposure day-7 either acclimated or not acclimated prior to exposure to treatments in mobile laboratory experiments conducted on Cape Cod Massachusetts in 2021 for Reference (REF), Fire-training area site-1 (FTA1), and Fire-training area site-2 (FTA2) treatments. See Table S2 for individual PFAS abbreviations.

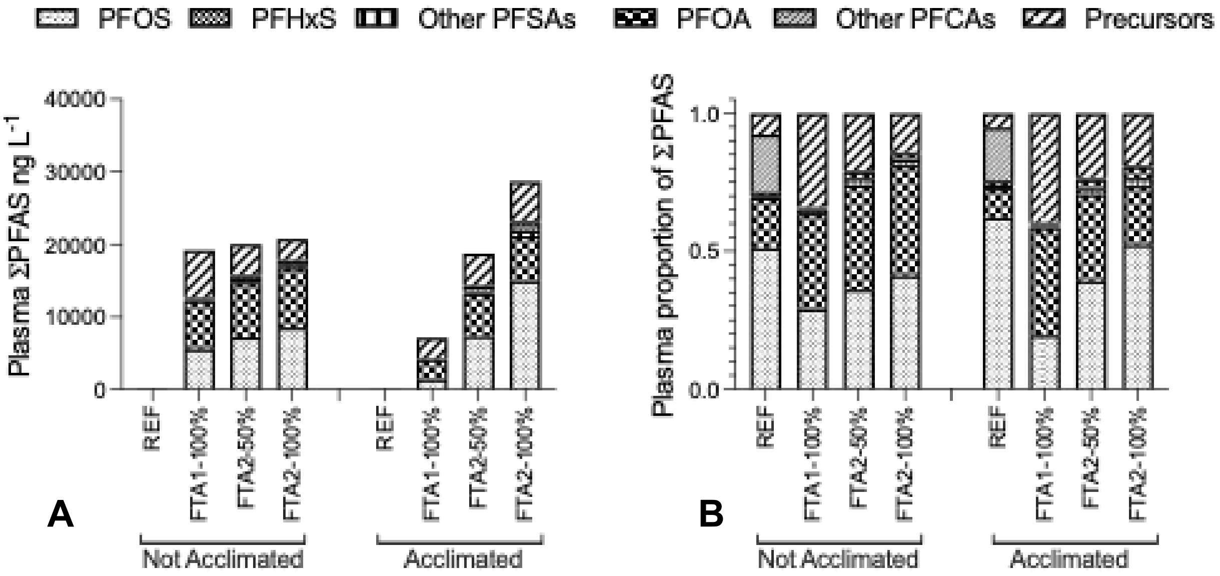

Figure S10. Plasma PFAS biotic concentration factor (CF_B, dimensionless) for perfluoroalkyl carboxylic acids (PFCA), perfluoroalkyl sulfonic acids (PFSA), fluorotelomer sulfonic acids (FTS) and perfluoroalkyl sulfonamides (FASA) on exposure day-7 in fathead minnow (*Pimephales promelas*) (A) not acclimated, or (B) acclimated prior to exposure to Reference (REF), Fire-training area site-1 (FTA1), and Fire-training area site-2 (FTA2) treatments in mobile laboratory experiments conducted on Cape Cod Massachusetts in 2021. See Table S2 for individual PFAS abbreviations.

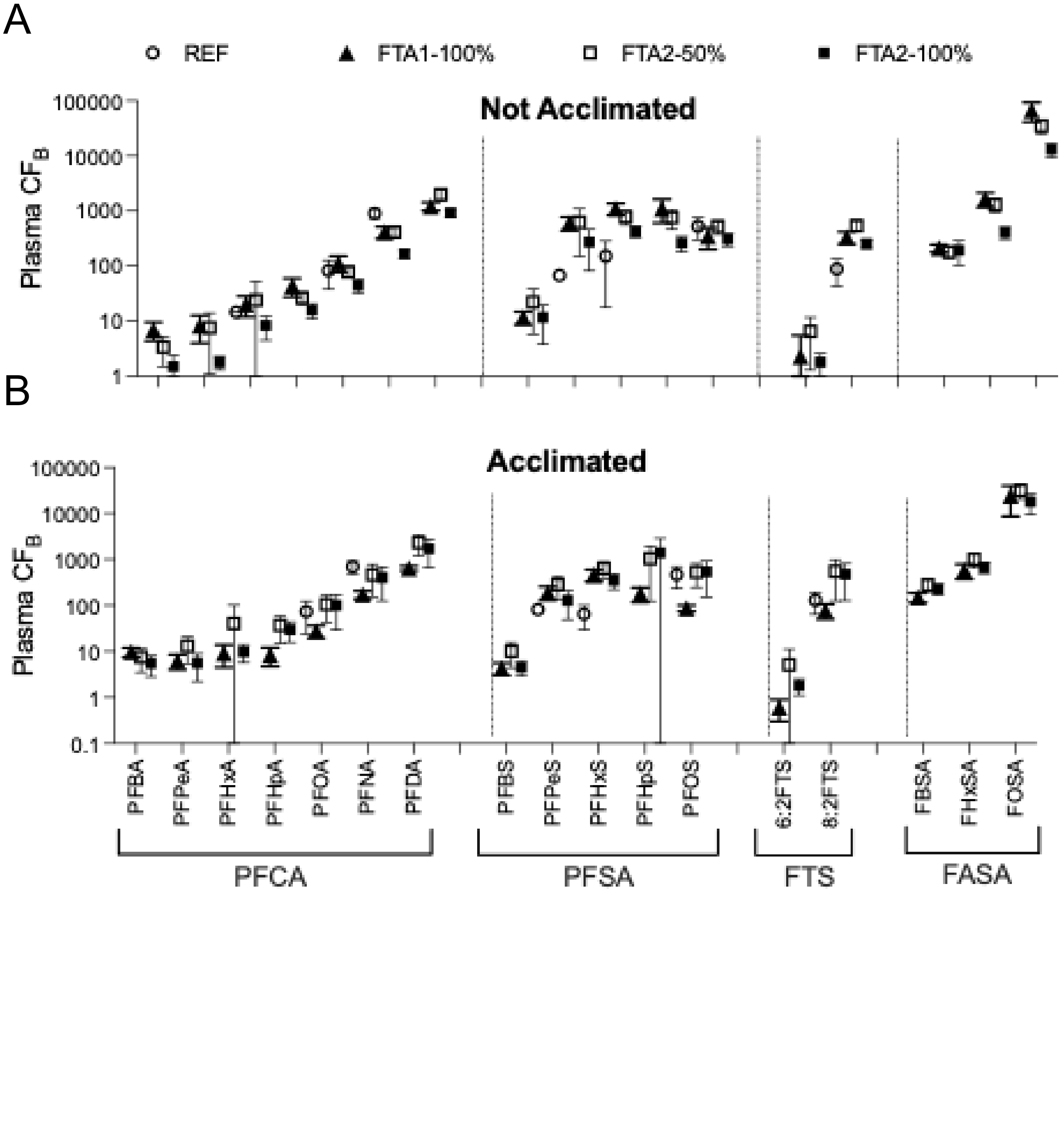

Figure S11. Cumulative mortality of male fathead minnows (*Pimephales promelas*) during 21-day exposure to Reference (REF), Fire-training area site-1 (FTA1), and Fire-training area site-2 (FTA2) treatments conducted on Cape Cod Massachusetts in 2018, 2019, and 2021. In 2021, early mortality and abnormal swimming behavior occurred only in FTA2-100% and FTA2-50% treatments, primarily within the first seven days.

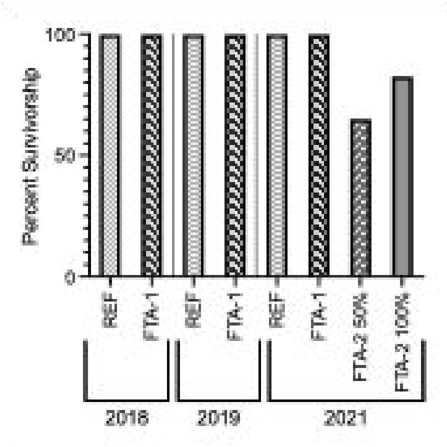

Figure S12. UpSet diagram summarizing nominally differentially expressed transcripts (p<0.05, unadjusted) across fathead minnow (*Pimephales promelas*) testis microarray comparisons for Reference (REF) and Fire-training area site-2 (FTA2) treatments. The matrix highlights shared and unique transcriptional responses and supports pathway-level comparisons among treatments.

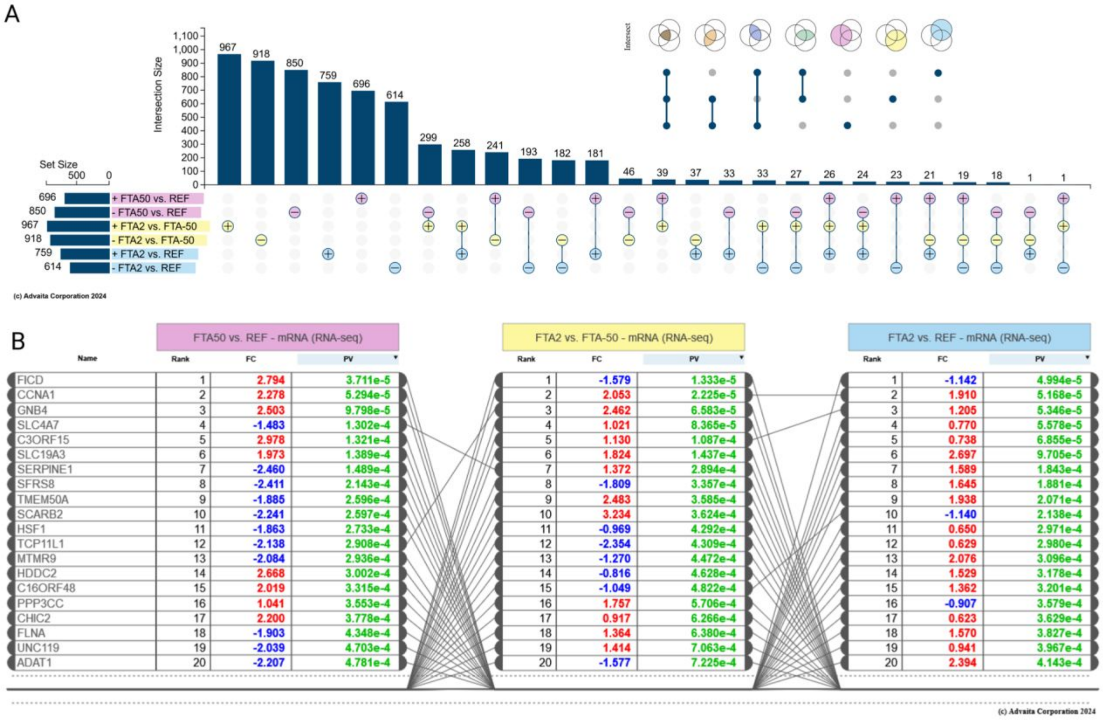

Figure S13. Hallmark signature pathways identified as enriched in the testis of fathead minnows (*Pimephales promelas*) exposed to Fire Training Area-2 (FTA2) 100% vs. Reference (REF) treatments, visualized with a chord diagram. Links indicate transcripts contributing to each signature pathway based on pathway enrichment analysis.

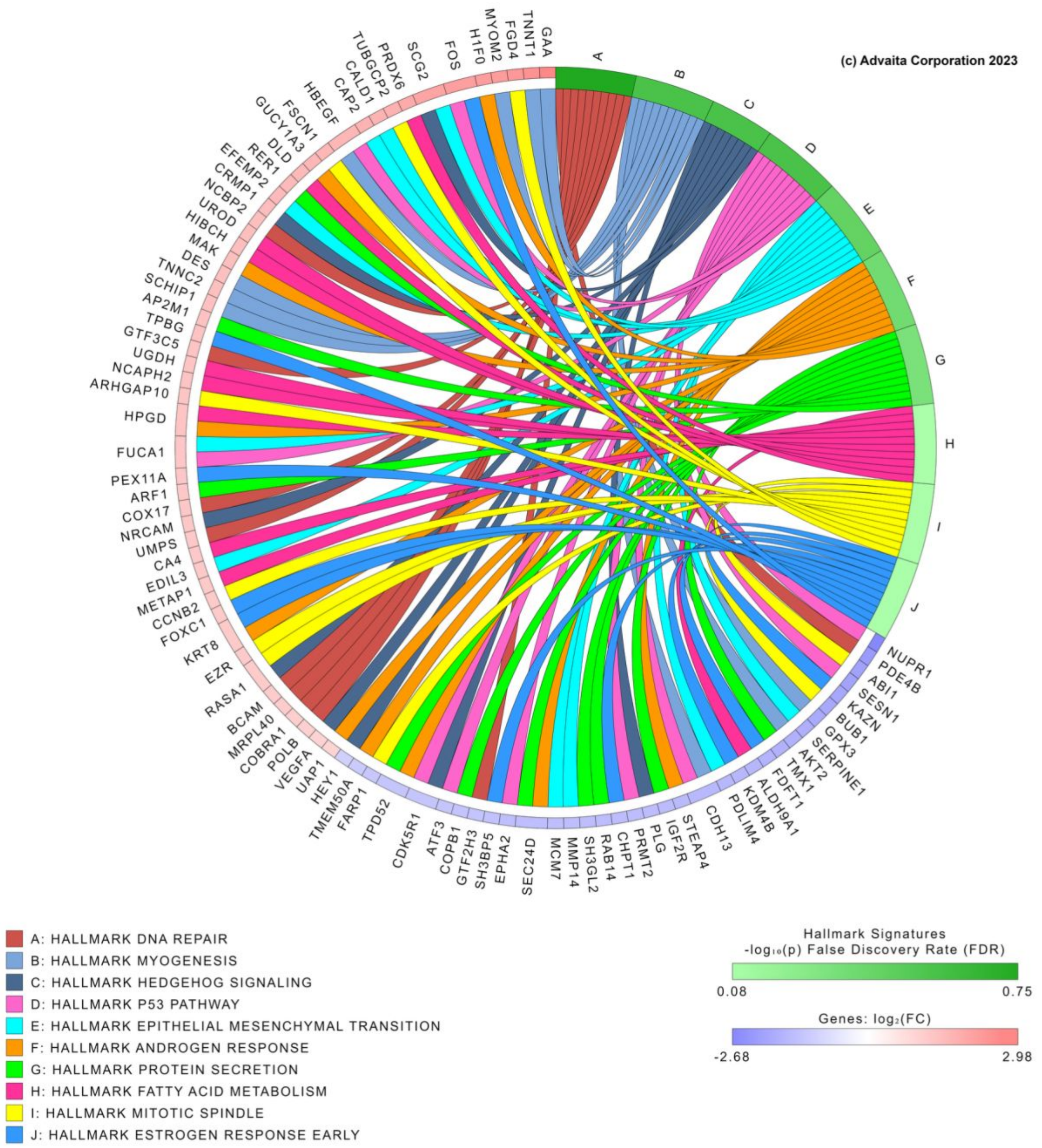

Figure S14. Pathways enriched in the testis of fathead minnows (*Pimephales promelas*) in the Fire Training Area-2 (FTA2) 100% vs. Reference (REF) treatments. Links indicate transcripts contributing to each signature pathway based on pathway enrichment analysis.

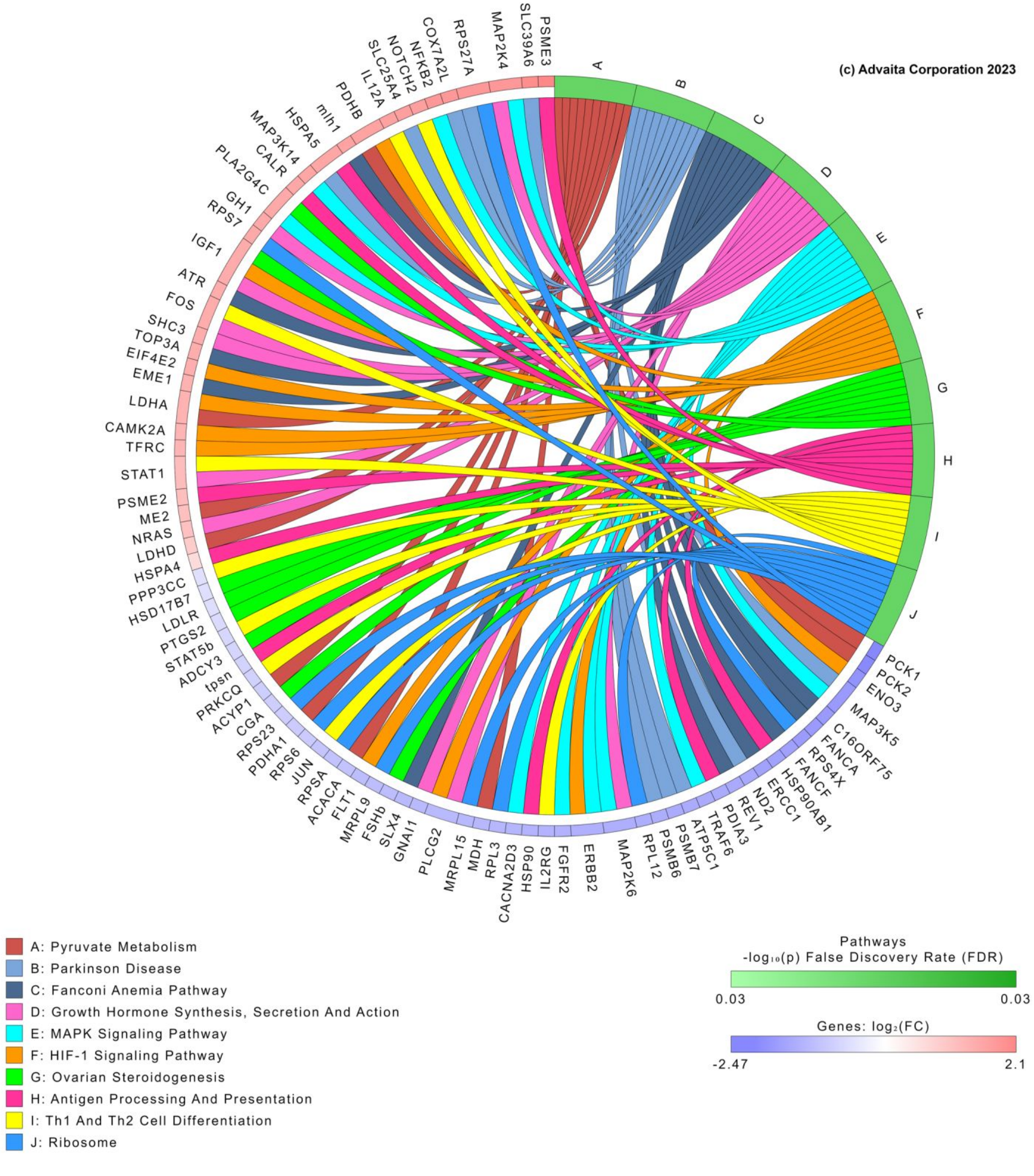

Figure S15. The RIG pathway was enriched in the testis of fathead minnows (*Pimephales promelas*) exposed to Fire Training Area-2 (FTA2) 50% vs. Reference (REF) treatments. Red arrows indicate pathway connections. Several downstream components show decreased expression in FTA2-50% relative to REF, consistent with treatment-associated modulation of this signaling network.

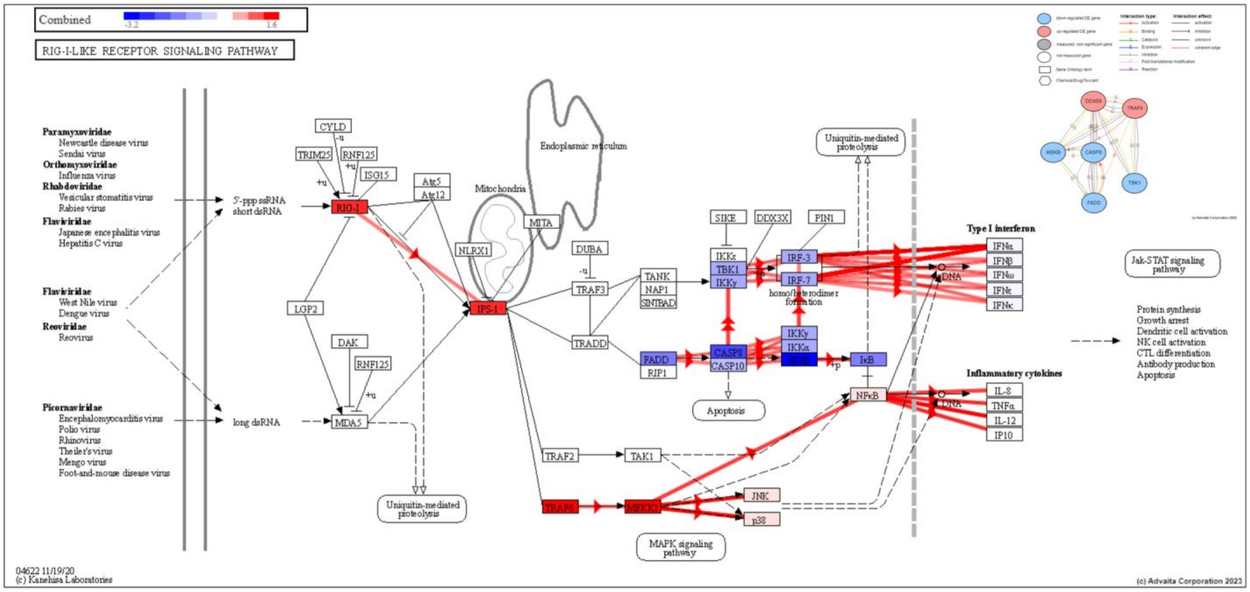

Figure S16. The Toll-like Receptor Pathway was enriched in the testis of fathead minnows (*Pimephales promelas*) exposed to Fire Training Area-2 (FTA2) 100% vs. Reference (REF) treatments. Red indicates upregulated transcripts and blue indicates downregulated transcripts.

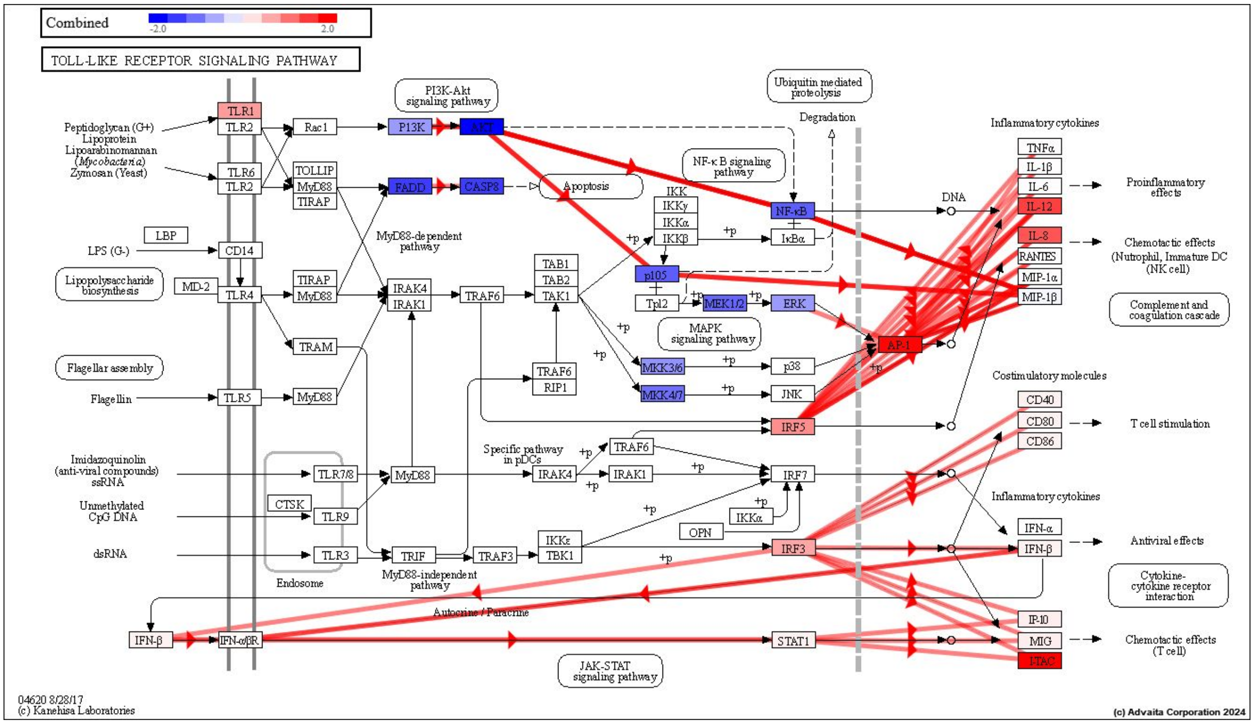

Figure S17. Testis transcriptomic responses in Fire Training Area-2 (FTA2) 100% vs. Reference (REF) treatments in 2021. (A) Overlap of nominally different transcripts (unadjusted $p < 0.05$) in testis microarrays from REF-acclimated male fathead minnows (Pimephales promelas) following 7-day exposures to FTA2-50% and FTA2-100% groundwater relative to REF, and between the two FTA2 treatments. No individual transcripts met false discovery rate criteria; therefore, transcript-level results are interpreted as exploratory and emphasis is placed on pathway-level patterns (see SI Tables S19–S22). (B) Heat map summarizing shared and treatment-associated pathway enrichment related to mitochondrial function, innate immune signaling, and endocrine regulation.

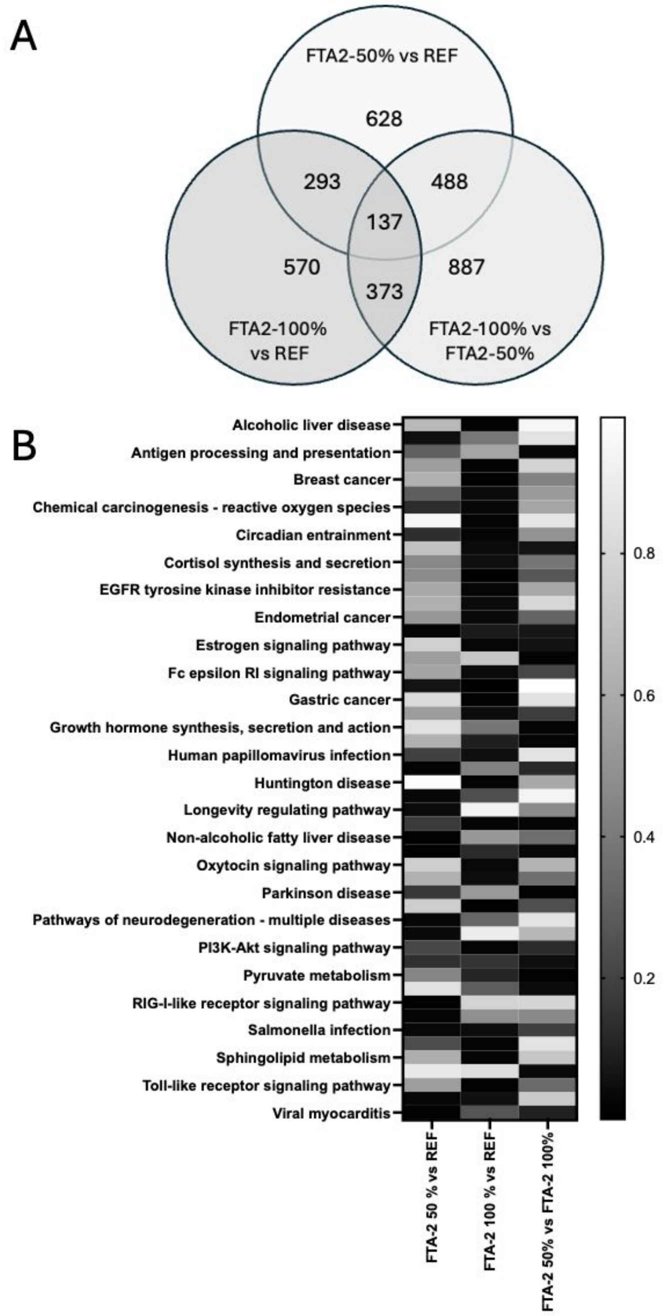

Figure S18. UpSet diagram summarizing nominally differentially expressed transcripts ($p < 0.05$, unadjusted) across fathead minnow (*Pimephales promelas*) liver microarray comparisons for Reference (REF), Fire-training area site-1 (FTA1), and Fire-training area site-2 (FTA2) treatments. Only annotated transcripts are shown. The matrix highlights shared and treatment-specific transcriptional responses and supports pathway-level comparisons among PFAS mixture exposures.

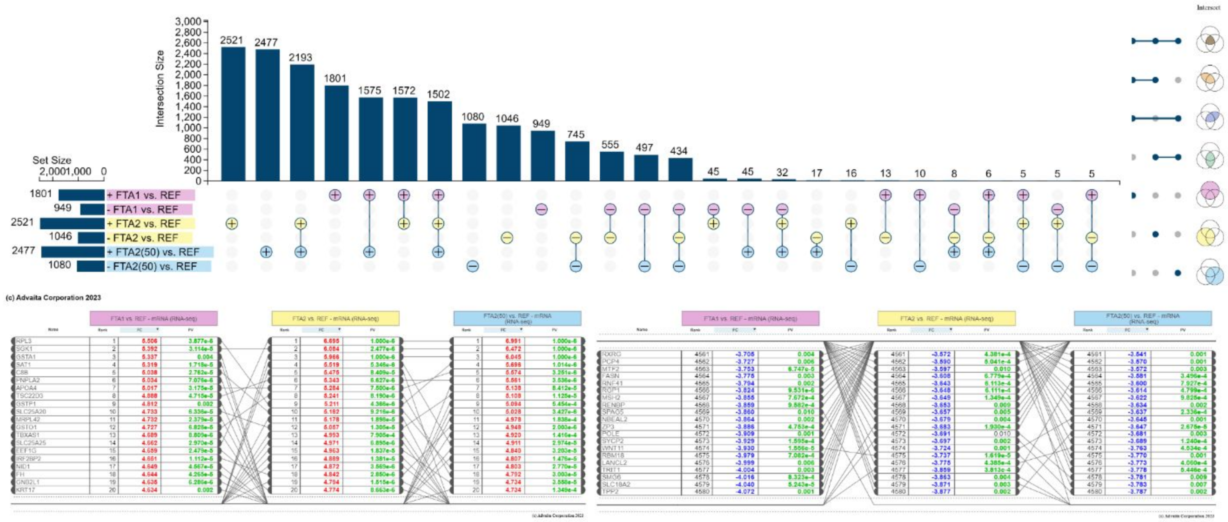

Figure S19. Hallmark signature pathways enriched in the liver of fathead minnows (*Pimephales promelas*) exposed to Fire Training Area-1 (FTA1) 100% relative to the reference site (REF), visualized using a chord diagram. Links indicate transcripts contributing to each enriched hallmark pathway based on pathway enrichment analysis.

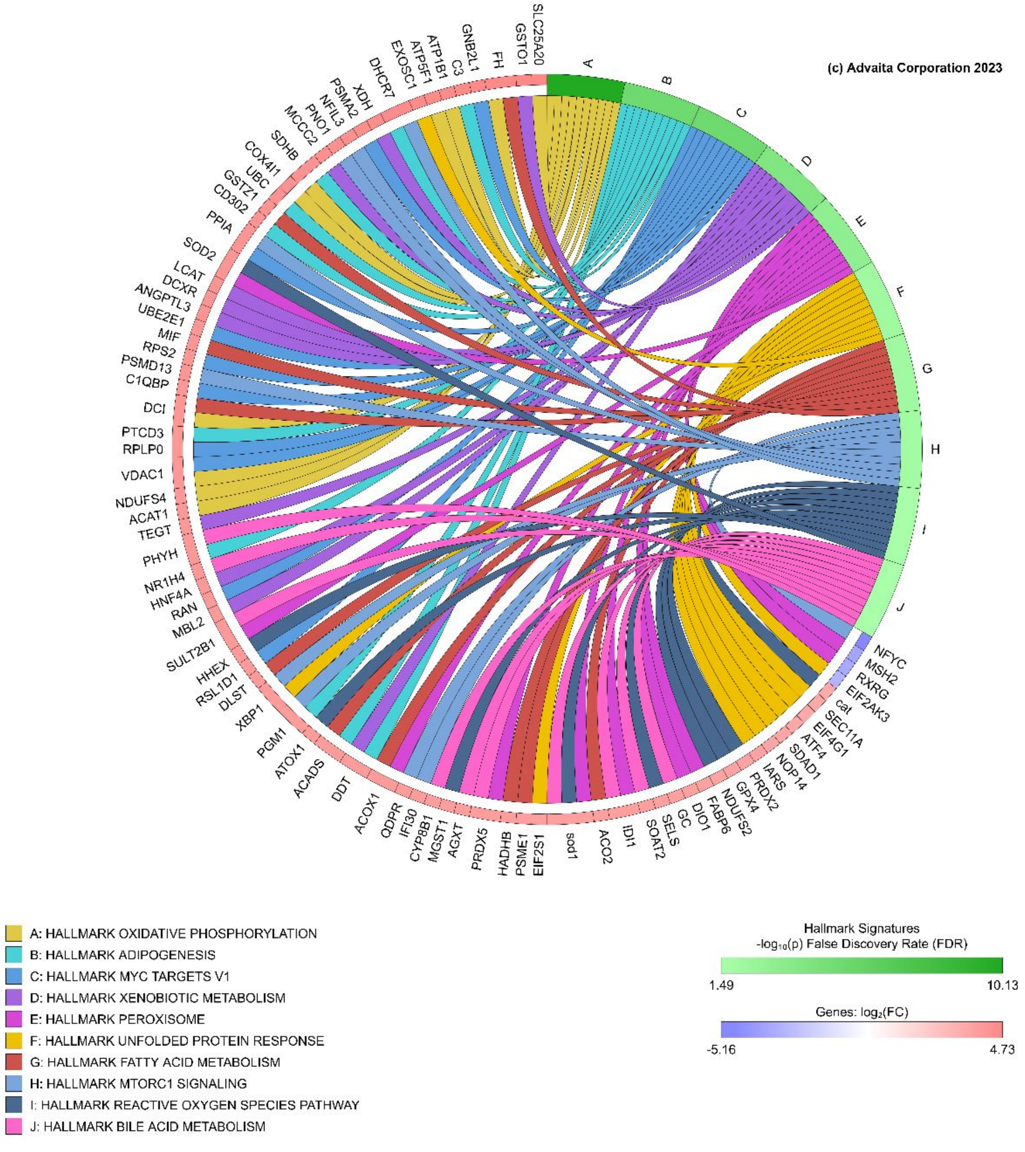

Figure S20. Hallmark signature pathways enriched in the liver of fathead minnows (*Pimephales promelas*) exposed to Fire Training Area-2 (FTA2) 100% relative to the reference site (REF), visualized using a chord diagram. Links indicate transcripts contributing to each enriched hallmark pathway based on pathway enrichment analysis.

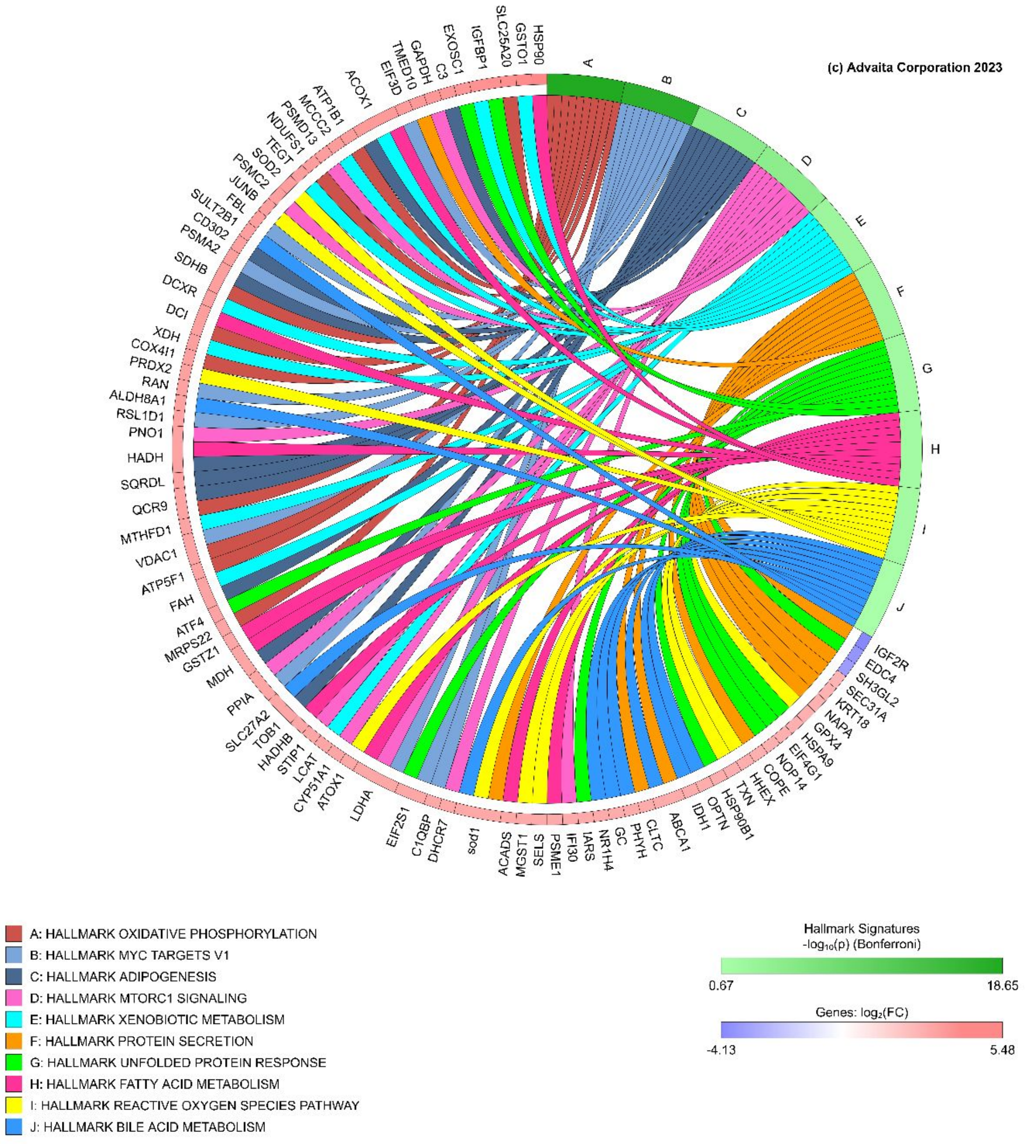

Figure S21. Oxidative phosphorylation (OXPHOS) deficiency pathway enriched in the liver of fathead minnows (*Pimephales promelas*) exposed to Fire Training Area-2 (FTA2) 100% relative to Reference (REF) treatments. Red indicates transcripts that were up-regulated relative to the reference condition.

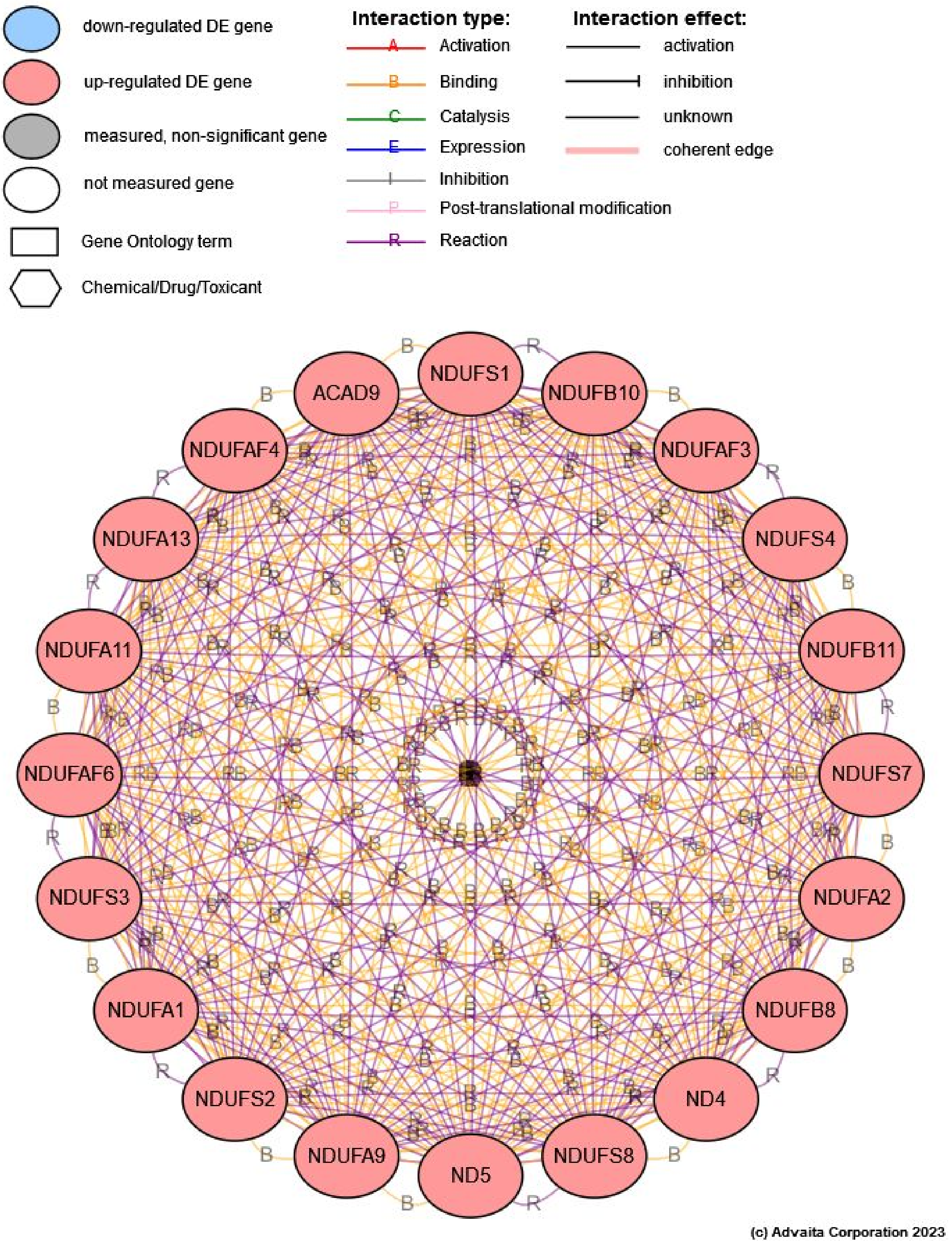

Figure S22. Estrogen signaling pathway enriched in the liver of fathead minnows (*Pimephales promelas*) exposed to Fire Training Area-1 (FTA1) 100% relative to the reference (REF) treatment. Several downstream components exhibit altered expression consistent with treatment-associated modulation of this signaling network. Red indicates increased transcript abundance and blue indicates decreased transcript abundance relative to REF.

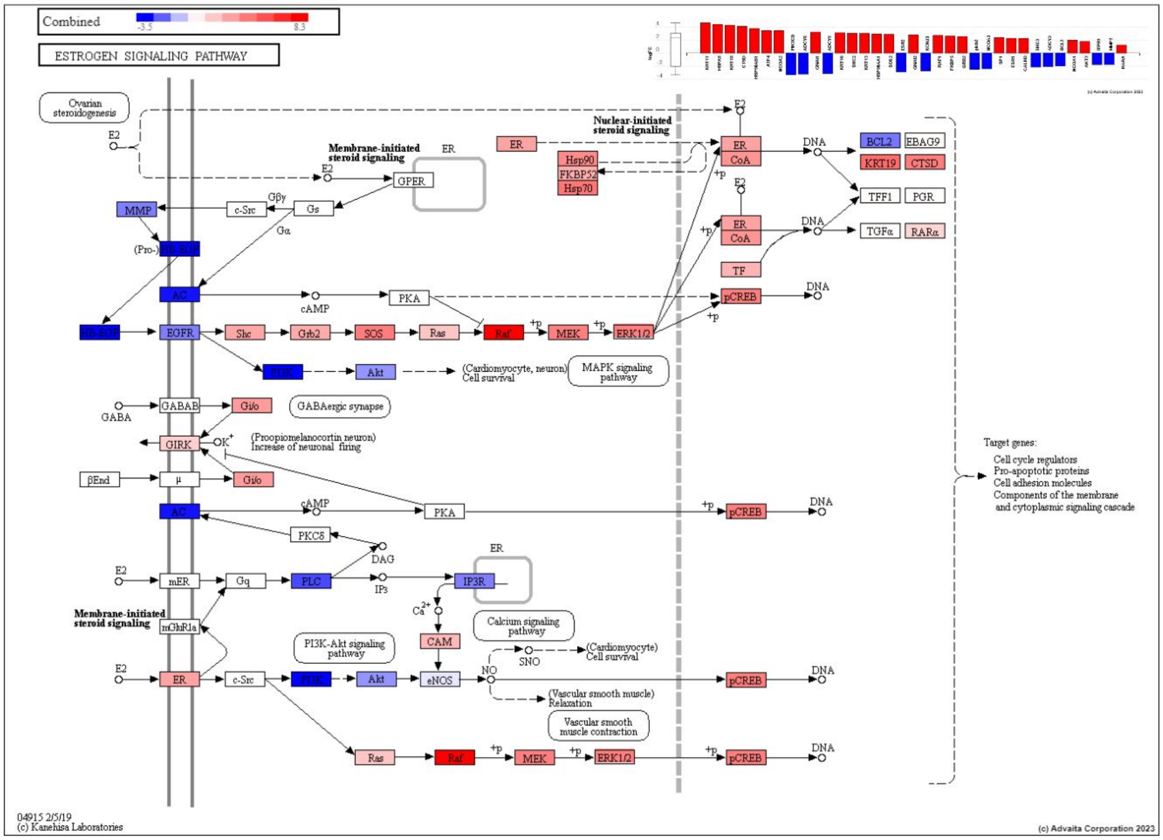

Figure S23. Predicted upstream chemical, drug, and toxicant signatures associated with liver differentially expressed genes (DEGs) in fathead minnows (*Pimephales promelas*) exposed to Fire Training Area-1 (FTA1) 100% relative to the reference (REF) treatment. The x-axis shows the fraction of overlapping DEGs with expression changes consistent with the predicted upstream signature (consistent/DE). Bubble size indicates the number of overlapping DEGs (# DE), and bubble color indicates enrichment significance as −log10(*p*).

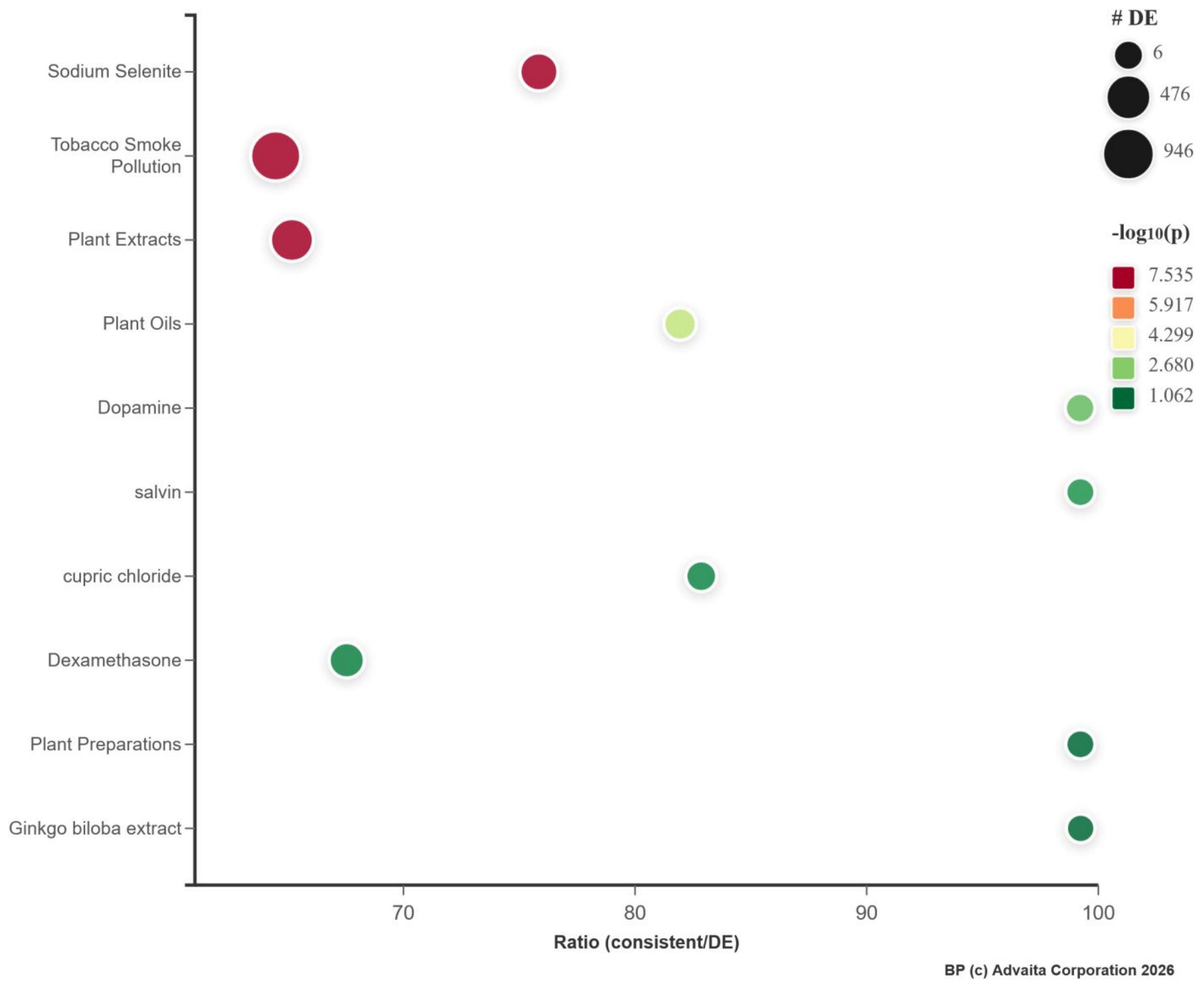

Figure S24. Predicted upstream chemical, drug, and toxicant signatures associated with liver differentially expressed genes (DEGs) in fathead minnows (*Pimephales promelas*) exposed to Fire Training Area-2 (FTA2) 100% relative to the reference (REF) treatment. The x-axis shows the fraction of overlapping DEGs with expression changes consistent with the predicted upstream signature (consistent/DE). Bubble size indicates the number of overlapping DEGs (# DE), and bubble color indicates enrichment significance as −log10(*p*).

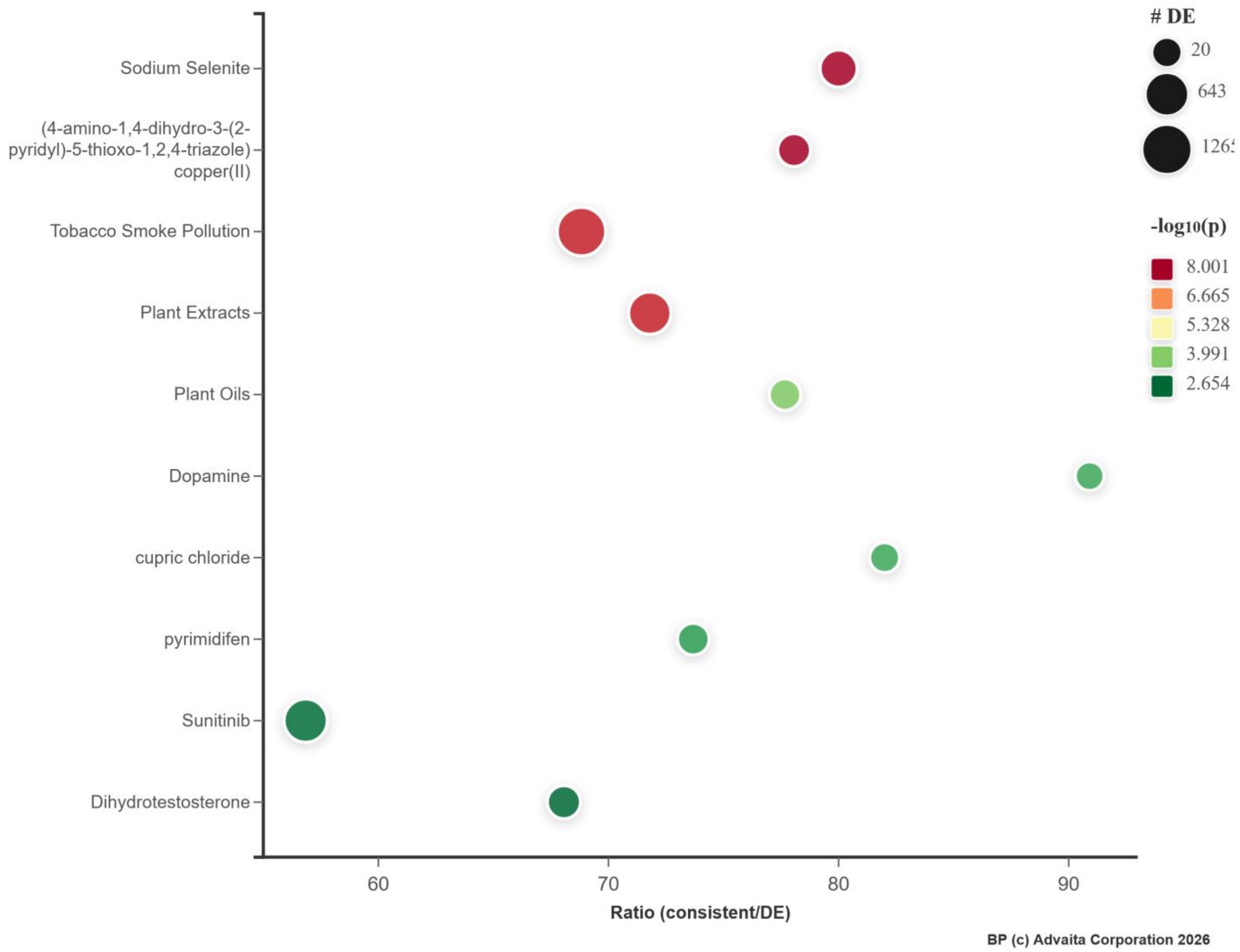

Figure S25. Predicted upstream chemical, drug, and toxicant signatures associated with liver differentially expressed genes (DEGs) in fathead minnows (*Pimephales promelas*) exposed to Fire Training Area-2 (FTA2) 50% relative to the reference (REF) treatment. The x-axis shows the fraction of overlapping DEGs with expression changes consistent with the predicted upstream signature (consistent/DE). Bubble size indicates the number of overlapping DEGs (# DE), and bubble color indicates enrichment significance as −log10(*p*).

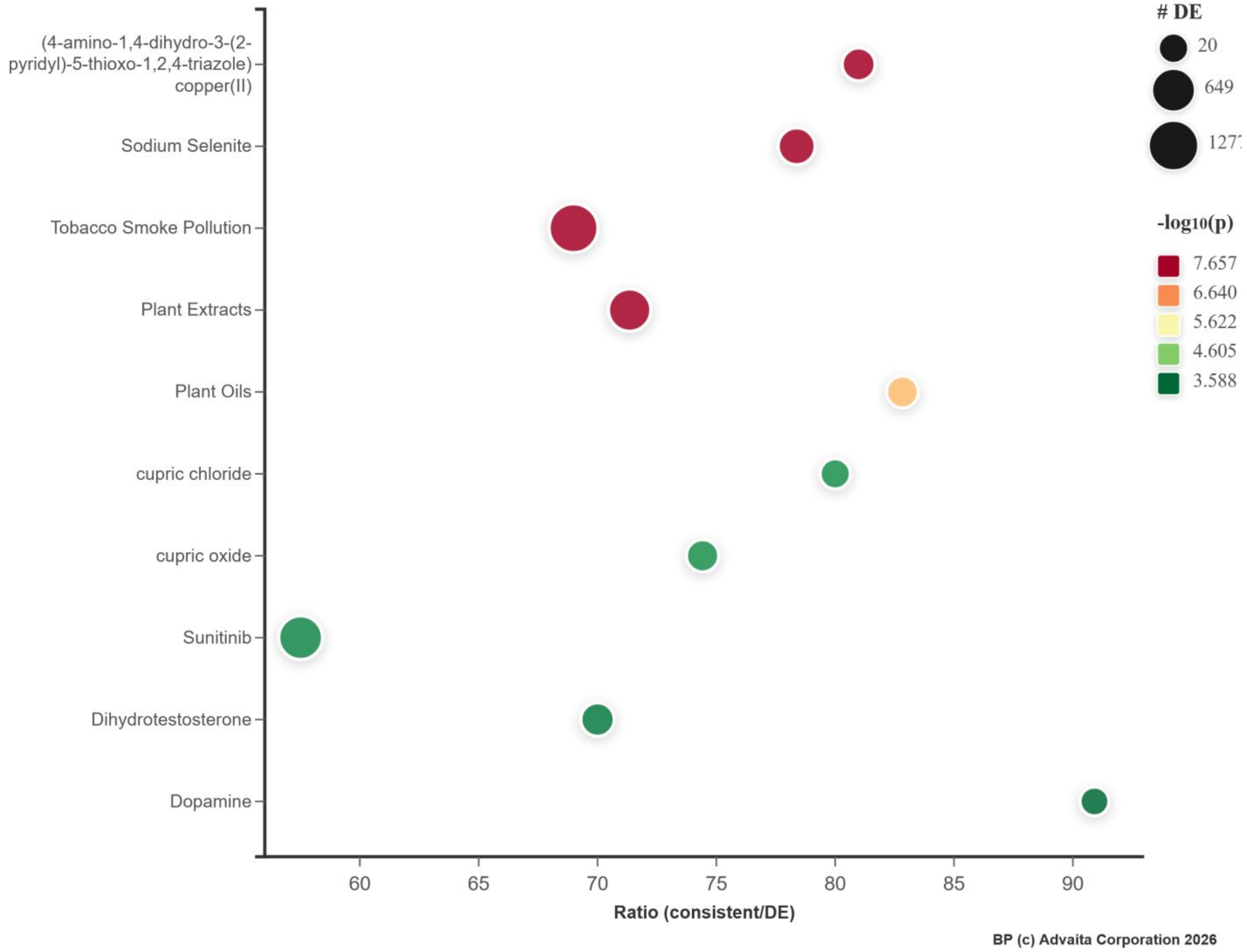

Supplement: Supplementary file 1 [file es6c01726_si_001.pdf]
